# Psychiatrists and non-psychiatrists’ attitudes to psychotropic optimisation for people with intellectual disabilities and/or autism: cross-sectional comparison study

**DOI:** 10.1192/bjo.2025.10875

**Published:** 2025-10-23

**Authors:** Samuel J. Tromans, Shoumitro Deb, Hassan Mahmood, Paraskevi Triantafyllopoulou, Tony Jamieson, Gill Gookey, Paul Bassett, Zayed Malak, Indermeet Sawhney, Laura Korb, Danielle Adams, Rory Sheehan, Rohit Shankar

**Affiliations:** SAPPHIRE Group, Department of Population Health Sciences, University of Leicester, Leicester, UK; Adult Learning Disability Service, Leicestershire Partnership NHS Trust, Leicester, UK; Imperial College London, London, UK; Coventry and Warwickshire Partnership Trust, Birmingham, UK; Tizard Centre, University of Kent, Canterbury, UK; NHS England, Nottingham, UK; Health Innovation East Midlands, Nottingham, UK; Hertfordshire Partnership University NHS Foundation Trust, Hatfield, UK; Barnet, Enfield and Haringey Mental Health NHS Trust, London, UK; University of Warwick, Coventry, UK; King’s College London, London, UK; University of Plymouth, Plymouth, UK; CIDER, Cornwall Partnership NHS Foundation Trust, Truro, UK

**Keywords:** Intellectual disability, autism, psychiatry, psychotropic, antipsychotic

## Abstract

**Background:**

Off-licence psychotropic use in people with intellectual disability and/or autism, in the absence of psychiatric illness, is a major public health concern in England.

**Aims:**

To ascertain and compare views of psychiatrists and non-psychiatrists working with people with intellectual disability and/or autism on psychotropic medication optimisation for this population.

**Method:**

A cross-sectional survey of 13 questions was disseminated online among psychiatrists and other health professionals working with people with intellectual disability and/or autism across England, using a non-discriminatory exponential snowballing technique leading to non-probability sampling. The questionnaire covered demographic characteristics, perceived barriers/benefits of psychotropic optimisation (including ethnicity) and views on implementation of a national medicine optimisation programme. Quantitative analysis used chi-squared, Mann–Whitney and unpaired *t*-tests, with significance taken as *P* < 0.05. Thematic analysis of free-text responses was undertaken with Braun and Clarke’s methodology.

**Results:**

Of 219 respondents, significant differences in attitudes to most issues emerged between psychiatrists (*n* = 66) and non-psychiatrists (*n* = 149). Psychiatrists had less optimism of a successful national medication optimisation programme if commissioned, or achieving 50% reduction in psychotropic overprescribing and inappropriate psychotropic prescribing generally. Perceived barriers to reducing overmedication differed significantly between the psychiatrists and non-psychiatrists, Thematic analysis identified five themes (system issues, resources, medication challenges, family and carers, and training and alternatives/structure).

**Conclusions:**

This is the first study to highlight important differences between psychiatrists and non-psychiatrists’ attitudes to psychotropic optimisation despite respondents overall being broadly supportive of its need. A major finding is the hitherto unquantified concerns of patient ethnicity and its impact on psychotropic optimisation principles.

Intellectual disability is characterised by significant limitations in both intellectual functioning and adaptive behaviour, with onset during the developmental period.^1^ The global prevalence of intellectual disability is approximately 1%.^2^ Autism is characterised by pervasive deficits in social interaction and communication, along with a pattern of restricted, repetitive behaviours, again with onset during the developmental period.^1^ The global prevalence of autism is approximately 0.8%,^3^ although there is a significant overlap of intellectual disability and autism,^4^ particularly for moderate to profound intellectual disability.^5^ People with intellectual disability and/or autism have significantly heightened rates of a wide range of physical and mental health conditions relative to the general population.^6,7^

The focus of this paper is on people with intellectual disability, particularly adults, who may or may not be autistic, but would collectively be termed people with intellectual disability and/or autism. This paper excludes able/high-functioning autism in its consideration.

## Psychotropic prescribing for people with intellectual disability

Around half (35–46%) of adults with intellectual disability receive psychotropics,^8^ primarily antipsychotics (27–35%), compared with <1% in the general population;^9^ however, the prevalence of psychosis is 2–4% in adults with intellectual disability, for which antipsychotics are primarily licenced. Long-term antipsychotic use increases the risk of serious adverse effects such as sedation, constipation, obesity, diabetes, and metabolic syndrome, which can impair a person’s quality of life and reduce life expectancy,^10,11^ risking hospital admission in some cases.^12^ In one study, 84.4% of 99 adults with intellectual disability who displayed challenging behaviour had at least one psychotropic-related adverse effect, and 45.6% had over three.^13^ Extrapyramidal symptoms were present in 53%, overweight or obesity in 46%, and metabolic syndrome in 11% of participants. Similarly, hyperprolactinaemia and one or more elevated bone turnover markers were present in 17 and 25%, respectively.^13^

In 2015, it was estimated that on an average day in England, between 30 000 and 35 000 people with intellectual disability are being prescribed an antipsychotic, an antidepressant or both without appropriate clinical indications.^14^ Additionally, antipsychotic medications are often prescribed to people without a recorded severe mental illness, but with a record of challenging behaviour, suggesting changes are needed in psychotropic medication prescribing for people with intellectual disability, and that further evidence of efficacy and safety of psychotropic medications is required for this patient group.^15^

Antipsychotic discontinuation continues to present a challenge; the proportion of participants among whom antipsychotics were discontinued completely increased in recent Dutch^19,20^ and UK^21^ studies compared with older UK studies.^22,23^ However, reinstatement of medication remains a problem, although the rate did improve in recent antipsychotic discontinuation studies.^17,24^ A survey among intellectual disability physicians, psychiatrists and specialist mental healthcare nurses attempted to gain insights into their experience in discontinuing long-term antipsychotics in people with intellectual disability with challenging behaviours.^35^ Complete cessation only occurred in a small fraction of cases, and impeding factors included relative, carer and support staff concern; lack of non-medication treatments and lack of multidisciplinary team (MDT) input. Previous work also reports that over half of patients (*n* = 106) with intellectual disability and challenging behaviour in a community intellectual disability team without a mental health diagnosis were prescribed psychotropic medications, with many of those having scarce MDT assessments to inform their care.^25^

Additionally, although some work has been undertaken to improve general practitioners’ knowledge on prescribing in people with intellectual disability,^26^ there is also a clear need for psychotropic medication management within specialist care.

The COVID-19 pandemic had an impact on people with intellectual disability. A survey^31^ showed that a large majority of carers noticed emotional changes in people they cared for. There were increases of 13% (2020) and 20% (2021) in regular psychotropic prescribing, and 21% (2020) and 24% (2021) had their ‘as required’ medication adjusted. People with intellectual disability were more likely to report being distressed compared with carers’ perception of their distress levels. However, such prescribing patterns demonstrated considerable regional variation; a service evaluation project reported an overall increase in psychotropic prescribing during the COVID-19 lockdown in urban compared with rural settings (11 *v.* 2%).^32^ A survey among clinicians in multiple nations^33^ showed increased clinician stress, referrals, presentations of patient distress, patient isolation and carer burden, and reduced participation of patients in activities of daily living. A third of respondents reported an increase in psychotropic medications.

For people with intellectual disability in England, antiseizure medications are now the second most widely prescribed psychotropic medication, with a significant portion of prescribing happening out of indication (i.e. for seizures).^29^ Prescribing of multiple psychotropic medications is prevalent in nearly half of people with intellectual disability who are prescribed antiseizure medications.^29^ Evidence of antiseizure medication benefit in management of challenging behaviours is limited. A review focusing on antiseizure medications prescribed for people with intellectual disability with a history of epilepsy indicated a reluctance to withdraw these medications.^30^

## The STOMP/STAMP programme and its impact

The overmedication of psychotropics in people with intellectual disability is a significant public health concern that has been addressed through the NHS England STOMP (Stopping Over-Medication of People with Learning Disabilities, Autism or Both (learning disability is the term used in the UK for intellectual disability))/STAMP (Supporting Treatment and Appropriate Medication in Paediatrics) initiative of medicine optimisation.^10^

Although the NHS England STOMP/STAMP programme has aimed to reduce inappropriate prescribing,^16^ there had initially been an absence of strong evidence on how best to reduce or stop psychotropic medications, which necessitates an MDT approach, consideration of co-occurring conditions and patient/carer involvement.^17,18^

Bespoke STOMP training for specialised intellectual disability teams has demonstrated that greater STOMP knowledge and understanding of best practice and guidelines is associated with training.^27^ It has been suggested that there is local development of STOMP training with national foundations for all specialist intellectual disability teams.

Since STOMP commenced, there have been changes in prescribing patterns. Antidepressants have replaced antipsychotics as the most widely prescribed antipsychotic among people with intellectual disability.^28^ The most likely reasons for the increase in antidepressant prescribing are for indications other than depression and increasingly extended treatment after symptom remission.

An online survey^34^ of UK psychiatrists working with people with intellectual disability to assess the impact of STOMP/STAMP on the psychiatrists’ practice showed that half of the 88 respondents stated that they started withdrawing antipsychotics over 5 years ago, and 52.3% said that they are less likely to initiate an antipsychotic since the launch of STOMP. However, since then, 46.6% prescribed other classes of psychotropic medication instead of antipsychotics for behaviours that challenge – most frequently, antidepressants. A complete antipsychotic discontinuation in over 50% of patients treated with antipsychotics was achieved by only 4.5% (*n* = 4) of respondents, but 11.4% reported deterioration in behaviour in over 50% of patients upon withdrawal, and the same proportion (11.4%) reported no deterioration.

An online questionnaire survey of 88 UK psychiatrists working with people with intellectual disability included two open-ended questions about the challenges psychiatrists faced locally to implement STOMP, and asked for examples of successes and positive and negative experiences from the process, including barriers to STOMP implementation.^36^ The qualitative analysis of free-text data showed variation within services in the experience and views of the psychiatrists. In areas with good support for STOMP implementation, psychiatrists reported satisfaction with the process with successful medication optimisation, increased local multidisciplinary and multi-agency working, and increased awareness of STOMP issues among the stakeholders such as people with intellectual disability, their caregivers and MDTs, and improved quality of life caused by reduced medicine-related adverse events in people with intellectual disability. However, where resource utilisation is not optimum, psychiatrists seemed dissatisfied with the process with little success in medicine optimisation. However, the survey only considered the views of psychiatrists, and did not capture the views of healthcare professionals from other professional backgrounds who also play a central role in supporting people with intellectual disability.

The aim of this study was to understand the views of psychiatrists and non-psychiatry healthcare professionals working with people with intellectual disability in relation to psychotropic optimisation.

## Method

The Strengthening the Reporting of Observational Studies in Epidemiology (STROBE) checklist (Supplementary File 1 available at https://doi.org/10.1192/bjo.2025.10875) was used to design and execute this cross-sectional study. The survey questions (Supplementary Files 2 and 3) were developed collaboratively by the authors. The survey was developed on the Microsoft Forms platform for Windows; the average completion time was approximately 10 min and was online from 3 to 20 October 2024. The survey questionnaire consisted of 13 questions related to the demographic characteristics of the respondents (e.g. job role, region in which they work), their views on implementation of a national quality improvement programme to reduce overprescribing and inappropriate prescribing, barriers that they had encountered with regard to medicine optimisation, benefits of medicine optimisation and strategies that they felt would help achieve this aim.

Participants of the study were psychiatrists and non-psychiatry health professionals working with people with intellectual disability. A non-discriminatory exponential snowballing technique leading to non-probability sampling was used to disseminate the survey in electronic form. The survey was disseminated via a convenience sampling approach, including through local professional networks, NHS England networks and the National Learning Disability Professional Senate.

### Analysis

#### Quantitative

The survey data from all participants was summarised descriptively. Additional analyses split the respondents into two groups based on job title of psychiatrists and non-psychiatrists and compare the responses between them. Categorical variables were compared between groups with the chi-squared test if there was no order to the categories. The Mann–Whitney test was preferred for categorical variables with a natural order to the categories. The unpaired *t*-test was used to compare continuous variables between staff groups. Significance was accepted at *P* < 0.05.

#### Qualitative analysis

Participants were invited to provide free-text responses to two survey items: one addressing the challenges of implementing psychotropic optimisation in special populations, particularly ethnic minorities, and the other focusing on strategies to rationalise psychotropic prescribing for people with intellectual disability and/or autism. Participants were also asked to provide their opinions on how the impact of these strategies could be measured. Responses were uploaded to NVivo 14 for Windows (Lumivero, Denver, CO, USA; see https://techcenter.qsrinternational.com/Content/nv14/nv14_standard_installation.htm) and thematically analysed (by P.T. and Z.M.), following Braun and Clarke’s methodology.^37^

## Results

### Quantitative findings

#### General findings

A total of 219 individuals responded to the survey. Table 1 summarises the characteristics of survey participants. With respect to job role, the largest respondent groups were psychiatrists (*n* = 66; 31%) and nurses (*n* = 66; 31%), followed by pharmacy staff (*n* = 27; 13%) and psychologists (*n* = 25; 12%).

**Table 1:** Demographic information for survey participants

Question	*n*	Category	*n* (%)
Job role	215^a^	Nurse	67 (31)
		Psychiatrist	66 (31)
		Psychologist	25 (12)
		Pharmacy Staff	27 (13)
		Social care professional	10 (5)
		General practitioner	4 (2)
		Other	16 (7)
Region	219	East Anglia	1 (<1)
		East of England	1 (<1)
		London	27 (12)
		Midlands	48 (22)
		North East	43 (20)
		North West	23 (11)
		South East	18 (8)
		South West	19 (9)
		Yorkshire	2 (1)
		Northern Ireland	3 (1)
		Wales	25 (11)
		Channel Islands	1 (<1)

a. Four respondents did not report their job role.

Participants were also asked to provide their views relating to reducing the prescribing of psychotropics; their responses are summarised in Table 2. The figures are the number and percentage in each category for the category variables, and the mean and standard deviation for continuous variables.

**Table 2:** Survey participant views on psychotropic prescribing

Question	*n*	Category	*n* (%)
Supportive of national quality improvement Programme	218	Very unsupportive	1 (<1)
		Unsupportive	0 (0)
		Unsure	13 (6)
		Supportive	50 (23)
		Very supportive	154 (71)
Can reduce overprescribing of psychotropics^a^	218	–	6.8 ± 2.2
Can reduce overprescribing of psychotropics (categorical scale)	218	Score 1–4	30 (14)
		Score 5–7	104 (48)
		Score 8–10	84 (39)
Encounter challenges implementing STOMP in ethnic minorities	219	No	62 (28)
		Don’t know	91 (42)
		Yes	66 (30)
Most important to reduce patient harm	207	Antidepressants	19 (9)
		Antiseizure	7 (3)
		Antipsychotics	60 (29)
		Benzodiazepines	24 (12)
		Combination psychotropics	88 (43)
		Hypnotics	9 (4)
Second most important to reduce patient harm	207	Antidepressants	19 (9)
		Antiseizure	14 (7)
		Antipsychotics	82 (40)
		Benzodiazepines	38 (18)
		Combination psychotropics	35 (17)
		Hypnotics	19 (9)
Least important to reduce patient harm	207	Antidepressants	53 (26)
		Antiseizure	73 (35)
		Antipsychotics	2 (1)
		Benzodiazepines	5 (12)
		Combination psychotropics	24 (12)
		Hypnotics	50 (24)
Importance of optimisation of psychotropics	219	Extremely unimportant	0 (0)
		Somewhat unimportant	1 (<1)
		Neutral	13 (6)
		Somewhat important	38 (17)
		Extremely important	167 (76)
Barriers to reducing inappropriate psychotropic use^b^	651	Family/carer preference	122 (19)
		Lack of access to alternatives	115 (18)
		No deprescribing guidance	42 (6)
		Lack of a multidisciplinary team approach	110 (17)
		Not a priority/focus	33 (5)
		Patient preference	28 (4)
		Primary care clinician capacity	19 (3)
		Primary care clinician confidence	54 (8)
		Psychiatrist capacity	46 (7)
		Psychiatrist confidence	33 (5)
		Unclear responsibility	49 (8)
Most important patient benefit from reducing Psychotropics	170	Increased social activity	11 (6)
		Reduced physical risks	84 (49)
		Reduced side-effects	73 (43)
		Increased activity	2 (1)
Least important patient benefit from reducing psychotropics	170	Increased social activity	68 (40)
		Reduced physical risks	9 (5)
		Reduced side effects	17 (10)
		Increased activity	76 (45)

STOMP, Stopping Over-Medication of People with Learning Disabilities, Autism or Both.

a. Summary statistics are mean ± s.d.

b. Each respondent gave up to three possible answers. % figures are percentage of all barriers listed (not % respondents).

There was widespread support for a national quality improvement programme, with almost three-quarters of survey participants being very supportive (*n* = 154; 71%), and 94% (*n* = 204) being either supportive or very supportive.

Participants were also asked whether they agreed that it is possible to significantly (>50%) reduce overprescribing or inappropriate prescribing of psychotropics in people with intellectual disability and/or autism on a 1–10 scale (10 representing the highest level of confidence in achieving this aim). The mean score was 6.8, with over a third (39%) scoring from 8 to 10 on the scale.

When asked if there were specific challenges in implementing psychotropic optimisation in ethnic minority communities, the most common response was ‘don’t know’ (*n* = 91; 42%). Of the other responses to this question item, roughly equal numbers selected the response items ‘yes’ (*n* = 66; 30%) and ‘no’ (*n* = 62; 28%).

When asked to rank the categories of medication that were most important to reduce patient harm, the most popular first choice was a combination of psychotropics, with 43% (*n* = 88) indicating that this was their first-choice medication. This was followed by antipsychotics, with 29% (*n* = 60) giving this as first choice, and an additional 40% (*n* = 82) indicating this to be second choice. Antiseizure medication was the least likely to be rated as first choice to reduce (*n* = 7; 3%), and was most likely to be ranked as last on the list (*n* = 74; 35%).

Almost all survey participants felt that it was important to optimise psychotropics, with 93% (*n* = 205) indicating that this was somewhat or extremely important. Only one respondent felt it was unimportant.

Participants were asked to give their top three barriers to reducing inappropriate psychotropic use. The most commonly reported barriers were family/carer preferences (*n* = 122; 19%), lack of access to alternatives (*n* = 115; 18%) and lack of an MDT approach (*n* = 110; 17%). The responses were not weighted.

The most frequently endorsed response options for the patient benefits from reducing psychotropics were reduced physical risks (49% of first-choice answers) and reduced side-effects (43% of first-choice answers). Conversely, increased social activity (6%) and increased activity (1%) were listed as the least important benefits.

#### Comparison between psychiatrists and non-psychiatrist groups

A subgroup analysis was conducted between psychiatrists (*n* = 66) and all other job roles (*n* = 149) (Table 3). Four respondents who did not state their job title were omitted from these analyses.

**Table 3:** Views on prescribing of psychotropics according to professional role

Question	Category	Psychiatrists, *n* (%)	Other roles, *n* (%)	*P*-value
Supportive of national quality improvement programme	Vey unsupportive	0 (0)	1 (<1)	**<0.001**
	Unsupportive	0 (0)	0 (0)	
	Unsure	9 (14)	4 (3)	
	Supportive	24 (36)	25 (17)	
	Very supportive	33 (50)	119 (80)	
Can reduce overprescribing of psychotropics^a^	–	5.7 ± 2.3	7.3 ± 1.9	**<0.001**
Encounter challenges implementing STOMP/STAMP in ethnic minorities	No	21 (32)	41 (28)	0.06
	Don’t know	14 (21)	76 (51)	
	Yes	31 (47)	32 (21)	
Most important to reduce patient harm	Antidepressants	8 (12)	11 (8)	0.12
	Antiseizure	0 (0)	7 (5)	
	Antipsychotics	16 (25)	42 (30)	
	Benzodiazepines	11 (17)	13 (9)	
	Combination psychotropics	29 (45)	57 (41)	
	Hypnotics	1 (2)	8 (6)	
Second most important to reduce patient harm	Antidepressants	8 (12)	11 (8)	0.23
	Antiseizure	5 (8)	8 (6)	
	Antipsychotics	30 (46)	52 (38)	
	Benzodiazepines	10 (15)	27 (20)	
	Combination psychotropics	5 (8)	28 (20)	
	Hypnotics	7 (11)	12 (9)	
Least important to reduce patient harm	Antidepressants	12 (18)	41 (30)	**0.01**
	Antiseizure	16 (25)	54 (39)	
	Antipsychotics	1 (2)	1 (1)	
	Benzodiazepines	1 (2)	4 (3)	
	Combination psychotropics	13 (20)	11 (8)	
	Hypnotics	50 (34)	27 (20)	
Importance of optimisation of psychotropics	Extremely unimportant	0 (0)	0 (0)	**0.04**
	Somewhat unimportant	0 (0)	1 (<1)	
	Neutral	2 (3)	11 (7)	
	Somewhat important	8 (12)	30 (20)	
	Extremely important	56 (85)	107 (72)	
Barriers to reducing inappropriate psychotropic use^b^	Family/carer preference	51 (26)	69 (16)	**<0.001**
	Lack of access to alternatives	43 (22)	68 (15)	
	No deprescribing guidance	13 (7)	29 (7)	
	Lack of a multidisciplinary team approach	35 (18)	74 (17)	
	Not a priority/focus	3 (2)	29 (7)	
	Patient preference	8 (4)	19 (4)	
	PC clinician capacity	3 (2)	16 (4)	
	PC clinician confidence	8 (4)	46 (10)	
	Psychiatrist capacity	18 (9)	28 (6)	
	Psychiatrist confidence	3 (2)	28 (6)	
	Unclear responsibility	10 (5)	38 (9)	
Most important patient benefit from reducing psychotropics	Increased social activity	2 (4)	8 (7)	0.20
	Reduced physical risks	22 (41)	60 (53)	
	Reduced side-effects	29 (55)	44 (39)	
	Increased activity	0 (0)	2 (2)	
Least important patient benefit from reducing psychotropics	Increased social activity	29 (55)	39 (34)	**0.009**
	Reduced physical risks	3 (6)	6 (5)	
	Reduced side effects	0 (0)	16 (14)	
	Increased activity	21 (40)	53 (46)	

STOMP, Stopping Over-Medication of People with Learning Disabilities, Autism or Both; STAMP, Supporting Treatment and Appropriate Medication in Paediatrics.

a. Summary statistics are mean ± s.d.

b. Each respondent gave up to three possible answers. % figures are percentage of all barriers listed (not % respondents).

Bold values indicate a significance of *P* < 0.05.

There was significant difference (*P* < 0.001) between groups for their support for a national medication improvement programme. Although both groups were generally supportive, the psychiatrists were less supportive than the other staff group. Half (50%) of psychiatrists (*n* = 33) were very supportive, compared with 80% (*n* = 119) of other staff.

There was also a significant difference (*P* < 0.001) in regard to the possibility of reducing the prescribing of psychotropics by >50%, with the psychiatrist group giving a mean score of 5.7, compared with 7.3 for the other staff group.

There was a significant difference (*P* = 0.01) for the least important medication with respect to reducing patient harm, where psychiatrists were more likely to indicate hypnotics and combination psychotropics as least important than the other group, whereas the other staff group was more likely to indicate antidepressants and antiseizure medication.

Importance of psychotropic medication optimisation were significantly different for the two groups (*P* = 0.04). Both groups felt that optimisation was important, but the psychiatrists felt it was more important: 85% (*n* = 56) of psychiatrists felt that this was extremely important, compared with only 72% (*n* = 107) of the other staff group.

The barriers to reducing inappropriate psychotropic use also demonstrated significant variation between the two staff groups (*P* < 0.001). Psychiatrists were more likely to indicate that family/carer preferences (*n* = 51; 26%) and lack of alternatives (*n* = 43; 22%) were a barrier than the other staff group. Responses for other staff were more evenly split between the different barriers.

There was a significant difference (*P* = 0.009) between the two groups for the least important benefit, with psychiatrists being more likely to indicate that increased social activity was least important (*n* = 29; 55%) compared with the other staff group (*n* = 39; 34%).

#### Qualitative findings

Participant responses regarding challenges in implementing psychotropic optimisation in special populations, particularly among ethnic minorities, were thematically analysed. Six themes were identified and are discussed below. All direct quotes for psychiatrists and non-psychiatrist professionals are presented in Supplementary File 4. Tables 4 and 5 capture the coding of psychiatrists and non-psychiatrists respectively with samples. Codes corresponding to quotes that feature in Tables 4 and 5 are included in brackets within the subsequent discussion of qualitative findings.

**Table 4:** Thematic analysis data table with examples of psychiatrist participant comments

	Themes and subthemes	Sample comments
1	*Staffing challenges and resource constraints*	
1.1	Staffing challenges	Adequate resources. Appropriate staffing, housing, social care input. Carers often have high turnover so any positive behaviour support or other measures can be difficult to embed. This can often lead to disturbances, which annul any benefit in medication reduction. Also, the drive for reducing in-patient admissions with inadequate resourcing of housing and caring from social care and the care service providers means that there is a backward step with any benefit from STOMP initiatives. For ethnic minorities, there have been situations where medication is seen as the only way to deal with issues rather than accepting, for example, there may be autistic challenges that require family to adapt and work with health professionals rather than family viewing the presentation as a brain defect that requires medication
1.2	Lack of effective MDT	Poor engagement from the MDT in implementing the much needed non-pharmacological interventions
1.3	Funding issues	Needs more funding for commissioning to provide more meaningful occupations and provision of respite care
1.4	Lack of proactive strategies	No proactive strategy in ensuring quality physical health checks are occurring
2	*Challenges with medication*	
2.1	Over-reliance on medication	Worsening of symptoms frequently leads to higher levels of medication
2.2	Behaviours that challenge	Medication sometimes is historically prescribed for behaviour and no additional diagnosis of mental illness was given. Communication approaches can be limited because of language barriers culture and norms can also have an influence as family often want a quick fix to challenging behaviours
2.3	Medication review	Access to reviews
2.4	Medical approach	Parents often come from abroad with children already prescribed high-dose antipsychotics, having apparently been told these will be needed very long term and to not reduce or stop them. This makes it difficult from the start. Additionally, many of these children have not been in school before coming to the UK and parents have not met with the concepts of behaviour as communication/sensory integration, or other ways of looking at behavioural presentations, and take a very medicalised approach. This is more a difference between children who have emigrated to the UK as opposed to different ethnicities whose children were born in the UK and have grown up with the ways of working here
3	*Working with carers, families and support staff*	
3.1	Resistance to change	Carer reluctance. Feeling that reduction of medication will be detrimental
3.2	Cultural barriers	Communication approaches can be limited because of language barriers, culture and norms
4	*Training and development of non-pharmacological approaches*	
4.1	Outcome measures	We need long-term outcome measures to highlight sustainability of reduction
4.2	New developments	We need to have alternatives/systems/resources/training in place to support the challenging behaviour that psychotropic medication was prescribed for
5	*Collaborative care structures and service-level support*	
5.1	MDT input	Do not start medication in the first place. We need MDT approach from day one. MDT approach, long-term funding options for community, robust staff teams that are appropriately enumerated financially for their training and expertise, full staff complement of community teams, prohibiting mental health detention/in-patient admission in the absence of community options

MDT, multidisciplinary team.

**Table 5:** Thematic analysis data table with examples of non-psychiatrist participant comments

	Themes	Sample comments
1	*Staffing challenges and resource constraints*	
1.5	Lack of skilled social care workforce	Services reducing and social care workforce that is not skilled enough
1.6	Instability in community settings	Hard to withdraw meds when people are struggling to be in stable settings
1.7	Non-pharmacological interventions	There is a real challenge with providing non-pharmacological interventions that are sustainable with infrastructure of learning disability services reducing and social care workforce that is not skilled enough and environments that are not capable
1.8	Financial constraints in staffing	Environment and not having financial facility to increase staffing can have an immense impact
2	*Challenges with medication*	
2.5	Over-reliance on medication	Families of ethnic minorities appear to see medication as the answer to fix behaviour and appear unaware that through support and using different strategies, they can make positive changes in their child’s behaviour. They appear to want a quick fix and seem to need more input around understanding the needs of their child and their behaviour
2.6	Over-prescribing	I do not find that there is an issue regarding ethnic populations – but general practitioners seem very happy to prescribe the intellectual disability population anything and everything – on occasion myself or my team have had to raise issues around contraindications of the medication prescribed.
3	*Working with carers, families and support staff*	
3.3	Resistance to change	Sadly I have experienced situations where parents have been extremely reluctant to have medication reduced and are often asking for more medication to be prescribed
3.4	Cultural barriers	When working with individuals who have immigrated to the UK, not enough is done to support the cultural change, and behaviours can be seen as psychological rather than exposure to a new culture/language/home
3.5	Impact of medication reduction on family and carers	People with a severe intellectual disability and in particular those prescribed first-generation antipsychotics. The reduction of antipsychotics in this cohort often leads to alleviation of sedation that can be perceived as challenging for carers and/or family
4	*Training and development of non-pharmacological approaches*	
4.3	Outcome measures	Outcome measures used to consider the impact of these approaches and psychological intervention on quality of life and engagement
4.4	New developments	Promote and offer alternative therapies such as positive behaviour support
5	*Collaborative care structures and service-level support*	
5.2	MDT input	Ensure all health checks have been completed. To consider least restrictive options and to weigh that up against risk. Discussions where appropriate with MDT members to support, assess and monitor. To ensure adequate support is in place for patient

MDT, multidisciplinary team.

### Theme 1: staffing challenges and resource constraints

All participants identified systemic barriers to implementing psychotropic optimisation initiatives. These included variability in practice across teams, inconsistencies in documentation and monitoring, and limited capacity for comprehensive reviews because of resource and organisational constraints.

Psychiatrist participants noted that inadequate resources, such as staffing, housing and social care input, make it difficult to embed non-pharmacological interventions like positive behaviour support. High staff turnover, particularly among carers, was seen to undermine stability in care, leading to behavioural escalations that make medication reduction more challenging. Additionally, the current focus on reducing in-patient admissions, without sufficient support infrastructure, was viewed as creating further setbacks. This can result in families, particularly those from ethnic minority backgrounds, relying on medication as the primary solution (subtheme 1.1).

Both psychiatry and non-psychiatry professionals stressed that these systemic issues are exacerbated by chronic underfunding. Psychiatrist participants especially emphasised the need for increased investment in services such as meaningful daytime activities, respite care and regular physical health checks, all of which are essential to reducing reliance on psychotropic medication (subthemes 1.3 and 1.4). Non-psychiatrist participants also described financial constraints within social care, noting that insufficient staffing and limited opportunities for training diminish the quality of care and hinder efforts to implement alternative interventions (subtheme 1.8).

At the level of day-to-day implementation, participants reported practical difficulties in delivering effective care under constrained conditions. MDTs often struggle to implement positive behaviour support and similar interventions when environments are not structured to support them (subtheme 1.2). Non-psychiatrist participants in particular noted the difficulty of supporting individuals who lack stable community placements (subtheme 1.6), and reflected on the broader impact of resource depletion across intellectual disability services, including insufficient numbers of trained staff (subthemes 1.5 and 1.7).

### Theme 2: challenges with medication

Both psychiatrist and non-psychiatrist participants face challenges related to medication use, with psychiatrists placing more emphasis on this issue. Participants highlighted an over-reliance on medication, noting a tendency to increase medication dosages when symptoms worsen (subtheme 2.1), They also emphasised the challenge of prescribing psychotropic medication for behavioural issues in the absence of a formal diagnosis of a mental health condition (subtheme 2.2). Language barriers further complicate effective communication, making it difficult for families and professionals to collaborate on treatment plans particularly for ethnic minority groups. Additionally, in some ethnic minority communities, cultural beliefs and systemic factors may influence families to view medication as a more accessible or acceptable approach for addressing behaviours that challenge, particularly when other forms of support are less readily available or culturally appropriate (subtheme 2.2).

A key challenge highlighted in psychiatry is the difficulty in conducting regular medication reviews (subtheme 2.3). Participants also noted that some parents – particularly within ethnic minority communities – may arrive with children or young people already prescribed high doses of antipsychotics. In some cases, these medications are viewed as long-term solutions, and families may express concerns about reducing or discontinuing them. This can present challenges when considering adjustments to treatment plans or exploring alternative approaches early on, especially when trust in services or access to culturally responsive care is limited (subtheme 2.4).

Non-psychiatrist participants also face challenges with an over-reliance on medication. Participants emphasised that medication is often seen as a quick solution for problems (subtheme 2.5). Families from ethnic minority backgrounds were reported to view medication as the primary solution for managing behaviour, often being unaware of alternative strategies that could positively influence their child’s behaviour (subtheme 2.5). Participants also mentioned that while not a specific concern regarding ethnic populations, General practitioners often prescribe medications liberally to people with intellectual disability and/or autism. In some cases, this has led to concerns regarding contraindications, necessitating intervention by the participants and their teams (subtheme 2.6).

### Theme 3: working with carers, families and support staff

Working with carers, families and support staff presents challenges for both psychiatrist and non-psychiatrist professionals. Both groups reported resistance to change and communication challenges, some of which were linked to cultural differences and misunderstandings in interactions with families from ethnic minority backgrounds. In psychiatry, participants highlighted carers’ reluctance to support medication reduction because of concerns about potential negative consequences for the individual (subtheme 3.1). Additionally, participants noted that differences in language and cultural norms can influence communication, which may affect the effectiveness of treatment and care (subtheme 3.2). Among non-psychiatrist professionals, participants described resistance from parents, who are often hesitant to reduce medication and may even request higher doses (subtheme 3.3). One participant discussed the challenge of reducing antipsychotic medications in people with severe intellectual disability, particularly those prescribed first-generation antipsychotics. Although reducing medication may alleviate sedation, it can also lead to the emergence of behaviours that may be perceived as challenging by carers and family members (subtheme 3.5). Participants also highlighted challenges when working with individuals who have immigrated to the UK. They noted that insufficient support for the cultural adjustment process often leads to behaviours that may stem from adapting to a new culture, language or environment being misinterpreted as psychological issues rather than understood as part of the adaptation process (subtheme 3.4).

### Theme 4: evaluating and developing non-pharmacological approaches

Outcome measures are a key focus for both psychiatrist and non-psychiatrist professionals in evaluating the effectiveness of psychotropic medication reduction. Psychiatrist participants emphasised the need for long-term outcome measures to assess the sustainability of medication reductions (subtheme 4.1). Similarly, non-psychiatrist participants highlighted the role of outcome measures in evaluating the impact of non-pharmacological approaches, such as psychological interventions, on quality of life and engagement (subtheme 4.3).

Additionally, participants in both professional groups stressed the need for new developments to support medication reduction. Among psychiatrist participants, this included the implementation of alternatives, systems, resources and training to manage any behaviours that challenge, for which psychotropic medications are often prescribed (subtheme 4.2). Non-psychiatrist participants advocated for the promotion of alternative interventions, such as positive behaviour support (subtheme 4.4). Both settings acknowledged the critical importance of non-pharmacological strategies in managing behaviours that challenge and reducing reliance on psychotropic medications.

### Theme 5: collaborative care structures and service-level support

Both psychiatrist and non-psychiatrist participants emphasised the importance of collaborative, multidisciplinary care structures in supporting the safe and sustained reduction of psychotropic medication. Psychiatrist participants highlighted the value of a strong MDT approach from the outset, noting that early and coordinated involvement of professionals can help prevent the unnecessary initiation of medication (subtheme 5.1). Participants also stressed the need for stable, long-term funding to ensure well-resourced community services, including adequately staffed teams and the full range of professional expertise. Without this foundation, efforts to reduce medication may be undermined. In particular, some participants raised concerns about the use of in-patient admissions or mental health detentions in the absence of robust community-based alternatives, which can disrupt continuity of care and escalate reliance on medication.

Non-psychiatrist participants similarly highlighted the need for comprehensive MDT input, emphasising the importance of the completion of all health checks and consideration of the least restrictive options alongside carefully balancing risk. Regular MDT discussions were identified as essential to support and monitor patients, ensuring that adequate systems and resources are in place to facilitate effective care (subtheme 5.2).

Table 6 highlights the main barriers reported by psychiatrist and non-psychiatrist participants to reducing inappropriate psychotropic medication use in people with intellectual disability and/or autism. These findings highlight common challenges encountered by both psychiatrists and non-psychiatrists, and reveal distinct differences in how these barriers manifest across settings.

**Table 6:** Most frequently reported barriers to reducing over or inappropriate psychotropic use among people with intellectual disabilities/autism

Barriers	Frequency in psychiatry (total *N* = 203), *n* (%)	Frequency in non-psychiatry (total *N* = 220), *n* (%)
Unclear clinical responsibility for ongoing prescribing	10 (5)	38 (17)
Psychiatrist capacity and confidence	20 (10)	57 (26)
Primary care clinician confidence	12 (6)	61 (28)
Lack of an effective MDT approach	36 (18)	80 (36)
Lack of deprescribing guidance	13 (6)	30 (14)
Lack of access to alternatives to medication	47 (23)	87 (39)
Family or carers preference	45 (22)	54 (24)
Carer or family or patient preference	6 (3)	20 (9)
STOMP/STAMP negative consequences	5 (2)	8 (4)
Lack of support	12 (6)	12 (5)
Diagnostic overshadowing	3 (1)	4 (2)
Medication reduction is not a priority focus	4 (2)	30 (14)

MDT, multidisciplinary team; STOMP, Stopping Over-Medication of People with Learning Disabilities, Autism or Both; STAMP, Supporting Treatment and Appropriate Medication in Paediatrics.

## Discussion

This survey reports the views of a substantial national sample of intellectual disability healthcare professionals (*N* = 219) about medicine optimisation. It is estimated that the survey captured the views of approximately 25–30% of all psychiatrists who work with people with intellectual disability and/or autism in England.

Participants reported widespread support for a national quality improvement programme to reduce inappropriate prescribing of psychotropics in people with intellectual disability and/or autism. Participants were also relatively optimistic regarding the possibility of achieving an >50% reduction in overprescribing or inappropriate prescribing of psychotropics in these patient groups. Furthermore, almost all participants viewed optimisation of psychotropic medication as important, with 93% (*n* = 205) indicating a view that this was either somewhat or extremely important.

However, when comparing the responses of psychiatrists (*n* = 66) versus non-psychiatrists (*n* = 149), important differences become evident. First, non-psychiatrists reported significantly more enthusiasm (*P* < 0.001) regarding a national quality improvement programme, and reported a significantly greater perceived achievability of a 50% reduction in overprescribing or inappropriate psychotropic prescribing (*P* < 0.001). The comparatively lower achievability view held by psychiatrists is somewhat supported by a systematic review by Costello et al^38^ on deprescribing interventions for adults with intellectual disability. The review highlighted that interventions aiming to reduce polypharmacy or deprescribe a single psychotropic medicine had ‘mixed success’.

However, seemingly in contrast to the enthusiasm of non-psychiatrists regarding a national quality improvement programme, they reported being less committed to the importance of psychotropic medication optimisation, although this finding only just met the threshold for statistical significance (*P* = 0.04). In the past 10 years, STOMP, despite outlining the need to reduce psychotropic prescribing in people with intellectual disability, has also been largely culpable in framing prescribing as a psychiatrist/doctor problem. This led to pressure on psychiatrists to reduce and stop medication without taking into account the large psychosocial and resource issues in the background influencing the problem. This could have led to weariness and scepticism from psychiatrists toward psychotropic medication optimisation programmes. Another concern is perceived therapeutic nihilism emerging from non-psychiatrists when applied to people with intellectual disability, where any prescribing of psychotropics is considered bad. This fails to take into account that people with intellectual disability have three times higher rates of mental illness than the general population, present to psychiatrists with distress and high risk, and prescribing is done on the balance of probabilities and harm reduction. There is a requirement to reduce ‘black and white’ thinking of medication, as psychotropic medication is justified for use in people with intellectual disability if it is suitable and rational. The current programme gives an opportunity to move away from these concerns of prescribing being a ‘doctor problem’ to a ’systems problem’, where one has to think of system balance from a biopsychosocial perspective to allow for optimum opportunities to reduce medication burden. Another possible explanation for this finding is some level of uncertainty regarding the definition of medication optimisation among non-psychiatrists, as a standard definition was not provided within the survey itself.

The barriers to implementing the psychotropic medicine optimisation programme STOMP mentioned by the participants in the current study, such as family and support staff’s concerns, lack of multi-agency and multidisciplinary input, and availability of non-medical psychosocial interventions, are similar to what was described by the UK psychiatrists in a recent online survey.^34^ Significant differences (*P* < 0.001) were also observed with regard to the perceived barriers to reducing overmedication, with psychiatrists most frequently reporting family/carer preference (*n* = 51; 26%), and non-psychiatrists most frequently reporting the lack of an MDT approach as a barrier (*n* = 74; 17%). These differences could reflect the differing professional experiences of these groups: psychiatrists may have clinical experience of families and/or carers expressing concerns about proposed reductions to a patient’s psychotropic medication regimen, whereas non-psychiatrist professionals may often feel left out of these discussions. These findings support those of a systematic review^39^ emphasising the importance of both stakeholder education and multidisciplinary collaboration in psychotropic deprescribing. Indeed, with respect to family/carer preference, a commentary^40^ has previously highlighted a tendency for clinicians and carers alike to opt for maintenance of the status quo, out of concerns of the potential adverse effects of psychotropic deprescribing. Patients themselves recognise the potential risks of psychotropic medication deprescribing, such as re-emergence of challenging behaviours.^36^

Our study respondents’ perception is that families with an ethnic minority background are over-reliant on medicine to address challenging behaviours. This is an under-researched area. There are also issues such as family cultural and educational background, stigma and family support networks to consider. Further, it is not clear if the identified ‘glass ceiling’ is attributable to clinician discomfort and training preventing them from broaching family matters/attitudes that may contribute to behaviours that challenge in people with intellectual disability and/or autism. Previous studies have shown that many families are over-reliant on medicine irrespective of ethnic background, and would like to see a more holistic approach to addressing behaviours that challenge.^41–43^ However, it is important to appreciate that the survey responses represent the views of respondents, and are not in themselves statements of fact. Indeed, the responses could indicate some level of unconscious bias among respondents, such as the perception of young black men as aggressive, and perhaps a need for training in this area.^44^ It may have also been helpful to collect data from survey respondents relating to their own ethnicity, to gauge the representativeness of the study population in this regard. We recognise ethnicity is a sensitive topic and should not be simplified, especially given that ethnic groups themselves have a range of sociocultural and economic factors influencing their attitudes. Nevertheless, our findings highlight a poorly recognised issue that has been having an impact on medication optimisation.

Factors such as lack of awareness of services, barriers to service access and different experiences of services are recognised as being contributory factors to health inequalities experienced by people with intellectual disability from ethnic minority backgrounds. Undertaking further research directly involving people with intellectual disability from different ethnic groups could help better explore whether those from ethnic minority groups do hold different views on the use of psychotropic medication, and if so, the underlying reasons.

Like the views of the participants in the current study, previous studies also showed a lack of psychotropic medicine knowledge among support staff and a need for appropriate training.^45,46^ To address the issue of support staff training, various resources have been created.

SPECTROM,^48^ an online, hybrid training resource with 14 modules and many internal and external resources, has been co-produced in the UK to help reduce the overmedication of people with intellectual disability and/or autism.^49^ Field trials in the UK and Australia showed that the training had improved support staff’s knowledge of psychotropic medicine and attitudes toward addressing behaviours that challenge without using medicine.^50,51^ The staff felt empowered to hold an informed conversation with the prescribers about psychotropic prescriptions.^50,52^

In 2019, Health Education England and NHS England published the ‘Core Capabilities Framework for Supporting People with a Learning Disability’. As part of this programme, five MindEd Learning Disability Mental Health modules comprising 14 separate sessions of e-learning were delivered.^52^ The sessions are intended to support the development of knowledge, skills and competencies related to the mental health, challenging behaviour and psychotropic prescribing of people with intellectual disability and/or autism.^53^ Similarly, Connect Behaviours is an interactive web-based tool designed to facilitate collaborative care planning and shared understanding to bring awareness, structure and evidence-based formulation to psychotropic optimisation.^54^ However, all of these initiatives lack a systematic national uptake because of the factors outlined in the paper.

### Strengths and limitations

This study collected clinically relevant survey data from a national population of intellectual disability healthcare professionals across a variety of professional groups. It provides important insights into the views of intellectual disability healthcare professionals at large, particularly on ethnicity matters, as well as the differences that exist between psychiatrists and non-psychiatrist groups.

The limitations of all cross-sectional studies, such as sampling bias, apply. Healthcare professionals that recognise the value of medicine optimisation may be more motivated to complete a survey on this topic relative to more sceptical colleagues. The questions related to people with intellectual disability and/or autism, and the survey was principally distributed through intellectual disability-related healthcare professional networks; professionals working only with autistic people without co-occurring intellectual disability, who represent a sizeable proportion of the autistic population,^4^ were actively excluded. Another group that are less represented in the participant population are general practitioners, who frequently support people with intellectual disability and/or autism not under the care of specialist services. Indeed, the majority of the study population comprised nurses (*n* = 67; 31%) and psychiatrists (*n* = 66; 31%), and one should be cautious about drawing firm conclusions about other professional groups, considering their comparatively small respective subgroup sizes. Previous national survey findings suggest that people with intellectual disability and/or autism encounter difficulties in both accessing and using primary care services,^6,7^ although less is known about their experiences specifically in relation to psychotropic medications.

Additionally, although the survey was developed collaboratively among professionals, people with lived experience of intellectual disability were not directly involved in its development, implementation or analysis. One aspect of the survey that could be considered a limitation is enquiring specifically about the challenges around implementing psychotropic optimisation in ethnic minority groups. Although there is some evidence to suggest that psychotropic optimisation is a particular challenge in members of certain ethnic minority groups, there is also a risk of this question potentially leading the respondent to think that this is an issue, particularly considering the absence of similar questions relating to other demographic groups, such as those based on age or gender. Related patient and public involvement work involving members of these communities could have helped inform as to whether this question should have been included in the survey, and if so, how it would be best phrased.

Furthermore, the survey was focused more on barriers than facilitators, and it is important to have a comprehensive understanding of both sets of factors; however, it is also essential for the surveys to not be overly lengthy to complete, which could discourage survey completion from all but the most highly motivated respondents. Finally, although the survey data shows important findings relevant to the care of people with intellectual disability and/or autism, it could have been supplemented with conducting semi-structured interviews among survey respondents, to better capture the complexity and nuance of the subject matter. Another approach would have been to increase the number of free-text question response items, but again, this may have deterred potential respondents from completing the survey.

### Implications for policy, research and clinical practice

It is important that there is sufficient buy-in from all healthcare professional groups working with people with intellectual disability and/or autism to effectively implement any proposed national quality improvement programme intended to reduce overprescribing and inappropriate psychotropic prescribing for this patient group. Furthermore, any interventions should endeavour to address the key barriers to deprescribing reported in this survey, such as lack of clarity regarding clinical responsibility, psychiatrist capacity and confidence, lack of an effective MDT approach, lack of deprescribing guidance, lack of alternatives to medication and carer/family/patient preferences. In particular, more insights are needed to be inclusive of ethnic minority groups.

In conclusion, although both psychiatrists and non-psychiatrist professionals are broadly supportive of medicine optimisation for people with intellectual disability and/or autism, important intergroup differences exist. Furthermore, this survey identifies frequently reported barriers to medicine optimisation. The ethnicity-related challenges outlined in this study is the perception of our respondents, which cannot be taken as evidence, but is an important aspect of understanding a hitherto poorly recognised problem. Any medicine optimisation intervention should endeavour to actively address such barriers to maximise its potential for success.

## Supporting information

Tromans et al. supplementary material 1 Tromans et al. supplementary material

Tromans et al. supplementary material 2 Tromans et al. supplementary material

Tromans et al. supplementary material 3 Tromans et al. supplementary material

Tromans et al. supplementary material 4 Tromans et al. supplementary material

## Data Availability

Data used for the study can be obtained from the corresponding author, R. Shankar, upon reasonable request.

## Supplementary material

The supplementary material is available online at https://doi.org/10.1192/bjo.2025.10875
