# Supplementary material for: Psychiatrists and non-psychiatrists’ attitudes to psychotropic optimisation for people with intellectual disabilities and/or autism: cross-sectional comparison study
Source: BJPsych Open. 2025 Oct 23;11(6):e249. doi: 10.1192/bjo.2025.10875 (PMC12569613; doi:10.1192/bjo.2025.10875)
Supplement: Tromans et al. supplementary material 1 — Tromans et al. supplementary material [file S2056472425108752sup001.docx]

| Themes | Participant comments (psychiatry) |
| --- | --- |
| 1. Breaking down barriers: Addressing systemic, structural, and resource challenges | |
| - 1. Staffing challenges | - Adequate resources. Appropriate staffing, housing, social care input. Carers often have high turnover so any PBS or other measures can be difficult to embed. This can often lead to disturbances which annul any benefit in medication reduction. Also the drive for reducing inpatient admissions with inadequate resourcing of housing and caring from social care and the care service providers means that there is a backwards step with any benefit from STOMP initiatives. For ethnic minorities there have been situations where medication is seen as the only way to deal with issues rather than accepting, for example, there may be autistic challenges that require family to adapt and work with health professionals rather than family viewing the presentation as a brain defect that requires medication. - Nursing shortage to monitor patient after reduction |
| 1.2 Professional barriers | - Psychiatry colleagues feeling threatened that if they reduce medication they will no longer be important. - Family, carers, or other health professionals' (like GP) reluctance to consider alternative options in lieu of medication. - Family and carer dependence, liaising with GPs |
| 1.3 Lack of effective MDT | - Lack of MDT availability to manage emergence of behaviours. - No evident engagement - Lack of timely MDT support - Poor engagement from the MDT in implementing the much needed non-pharmacological interventions. - Needs more funding for commissioning to provide more meaningful occupations and provision of respite care. - Service level gaps in professions secondary to long term vacancies, leaving the MDT incomplete. LA level cut backs put a huge pressure on the MDTs ability to provide person centred care. Ongoing lack of understanding related to STOMP initiatives in the MDT and wider public; resulting in preference for a 'quick fix'/the medical model. |
| 1.4 Lack of awareness | - Significant lack of proper awareness on what autism is & available resources in managing aspect of their mental health & from external agency - Ongoing lack of understanding related to STOMP initiatives in the MDT and wider public; resulting in preference for a 'quick fix'/the medical model. |
| 1.5 Delayed input | - Slow input from identified areas with referrals made to multidisciplinary professionals & external agency( social service). |
| 1.6 Lack of access to alternatives | - Lack of appropriate non pharmacological interventions - I work in an ethnically diverse area. I find that patients often get medications from abroad or dismiss non pharmacological options easily. - Absence of other interventions (eg: psychological) - extra "weight" placed upon role of medication rather than psychosocial/behavioural interventions - Poor engagement from the MDT in implementing the much needed non-pharmacological interventions. - people lack alternatives to medication in reducing risks from physical altercations particularly in staffed settings. - less likely for behavioural approaches to be implemented - No resources have been made available to deliver this programme |
| 1.7 Funding issues | - Needs more funding for commissioning to provide more meaningful occupations and provision of respite care. |
| 1.8 Lack of proactive strategies | - No proactive strategy in ensuring quality physical health checks are occurring. |
| 1.9 Resistance to change | - Carers reluctance. Feeling that reduction of medication will be detrimental. - resistance from family/care - People's expectations that medications are helpful, and so can be reluctant to withdraw. - Family, carers, or other health professionals' (like GP) reluctance to consider alternative options in lieu of medication - The proxy/family/carers have the unshakable belief that pharmacological intervention is the ultimate solution to the challenging behaviours displayed by their loved ones. - Carers unable to cope with small increases in behaviours. - Communication approaches can be limited because of language barriers Culture and norms can also have an influence as family often want a quick fix to challenging behaviours - People's expectations that medications are helpful, and so can be reluctant to withdraw. |
| 1.10 Cultural barriers | - Communication approaches can be limited because of language barriers Culture and norms - language barriers - I work in an ethnically diverse area. I find that patients often get medications from abroad or dismiss non pharmacological options easily. - Additionally, many of these children have not been in school prior to coming to the UK and parents haven't met with the concepts of behaviour as communication/sensory integration, or other ways of looking at behavioural presentations, and take a very medicalised approach. This is more a difference between children who have emigrated to the UK as opposed to different ethnicities whose children were born in the UK and have grown up with the ways of working here. - Getting the mdt and family and carers to support - Red tape from within team & external agencies, i.e oor communication amongst teams. - language barriers - communication and engagement - Communication approaches can be limited because of language barriers Culture and norms |
| 1.11 MDT input | - Timely input form MDT members - Much more timely access to nursing and MDT input around risk behaviours so this always goes in first before the referral to psychiatry happens. - Clear guidance from the RCPsych that bnf and nice must be followed and it does not condone idiosyncratic prescribing. Also can the RCPsych stop promoting use of ADHD medication and links of psychiatrist with drug companies. It is creating a new generation of patients that will require ADHD STOMP in the future. Has the college the ability to identify conflicts of interest? - Follow the very sensible RCPsych guidelines on this topic. - Better guidance, active involvement and leadership by NMP especially pharmacists - Ensuring good and holistic MDT input including psychology, behavioural support and social care input, - Strong MDT support with robust care providers who can offer support in appropriate environments. - need for more MDT approach - MDT approach and onboard carers - MDT input to intervention and joined up approach - consistent use of non medication approaches alongside the use of psychotropics where necessary - MDT approach, long term funding options for community, robust staff teams that are appropriately enumerated financially for their training and expertise, full staff complement of community teams, prohibiting mental health detention/inpatient admission in the absence of community options. - Don't start medication in the first place. Need MDT approach from day one . - regular medication review and MDT involvement - agreeing that medication are for short term use only until the MDT can work on a formulation - we need evidence based tools and suitable trained MDT resources such as pharmacists dedicated for this additional work not expect it to be part of already routine work - regular clinical reviews MDT working STOMP database with regular oversight and discussion support from specialist LD pharmacist |
| 1.12 identifying undiagnosed comorbid conditions | - A strategy to better 'diagnose' the comorbid conditions in the patient population - this could be measured by comparing diagnostic rates for the common comorbidities (ADHD/ASD/Bipolar/Schizophrenia/Schizoaffective/OCD/GAD/Depression) across populations. Until we have diagnostic rates of the above in our dataset that roughly match what is seen in the large meta-analyses our data is essentially meaningless to determine what is 'inappropriate prescribing'. - In my experience many patients on antipsychotics for example actually have a comorbid condition that has not been diagnosed - and the patient is stable as they are responding to medication. The biggest flaw in the current data is that it flags anyone (certainly the older datasets anyway) who has ID/ASD and is on medications and doesn't allow us to look at if they have a comorbidity that the medication may be treating. - Once we are confident that comorbid diagnoses are coded we can then more easily identify those on psychotropics who don't have diagnoses that may indicate them (ie risperidone has evidence for use in bipolar/psychosis/OCD/ADHD/PTSD/Tics/Treatment resistant depression - not as a first line agent for many of these conditions). This will be a far richer dataset to produce meaningful data and allow targeted interventions to the patient groups. |
| 1.13 Clear pathways for medication reduction | - he same person reviewing in clinic. This has its issues because trainee doctors may see the simpler 'stomp cases' but the problems with reduction in medication start only when they leave. This might be a role for specialist nurses to oversight or specialist pharmacists. There needs to be clear pathways and access to appropriate alternatives to medication via an appropriately resourced team - Evidence of less medication use, less side effects & better quality of life for patients - Start low and increase slowly, try to establishing routine, try higher dose to gain control, establish routine and slowly reduce medication. Reduction in frequency and intensity of behaviour, reduction in stress for carers. - This has its issues because trainee doctors may see the simpler 'stomp cases' but the problems with reduction in medication start only when they leave. This might be a role for specialist nurses to oversight or specialist pharmacists. There - Keeping a register of children prescribed the different medications locally and regularly reviewing this to look for prescribing patterns |
| 1.14 accurate diagnosis for medication prescription | - Give mental health diagnoses where appropriate when prescribing medication for behaviour - Concerns about prescribing the correct medication as there can be difficulty making the right diagnosis. Concerns about side effects and causing harm. - Concerns about prescribing the correct medication as there can be difficulty making the right diagnosis. - Identify disorder or symptoms that are being targeted by the psychotropic and ensure there is an evidence base supporting choice of Rx. - clarity around rationale for prescribing - Goal based approach to prescribing and de-prescrbing - Measuring outcomes following introduction of medications and de-prescribing if not effective - Avoid multiple simultaneous psychotropic medication introduction as well as de-presecribing - For patients who have been on long term medication who do not have capacity to consent to de-prescribing best interests meetings should happen for all parties to accept that deprescribing potentially presents a risk of destabilising significantly well-settled patients. |
| 1. Implementing STOMP in ethnic minorities | |
| 2.1 Over reliance on medication | - Worsening of symptoms frequently leads to higher levels of medication. - extra "weight" placed upon role of medication rather than psychosocial/behavioural interventions - Ongoing lack of understanding related to STOMP initiatives in the MDT and wider public; resulting in preference for a 'quick fix'/the medical model. - over reliance on medication less likely for behavioural approaches to be implemented |
| 2.2 Medication review | - Access to reviews |
| 2.3 Medical approach | - Parents often come from abroad with children already prescribed high dose antipsychotics, having apparently been told these will be needed very long term and to not reduce or stop them. This makes it difficult from the start. Additionally, many of these children have not been in school prior to coming to the UK and parents haven't met with the concepts of behaviour as communication/sensory integration, or other ways of looking at behavioural presentations, and take a very medicalised approach. This is more a difference between children who have emigrated to the UK as opposed to different ethnicities whose children were born in the UK and have grown up with the ways of working here. - extra "weight" placed upon role of medication rather than psychosocial/behavioural interventions |
| 1. Training and development of non-pharmacological approaches | |
| 3.1 Decision making | - More mental capacity Act advocates who can support best interest decision making |
| 3.2 outcome measures | - We need long term outcome measures to highlight sustainability of reduction - Measure - objective and subjective assessments of target behaviours, psychopathology, S/Es and QoL. - Could be measured looking at overall prescribing for a caseload over time with different interventions and parental attitudes to care. NB in my caseload children have moderate LD or lower and are generally unable to feedback themselves. - Mainly the direct feedbacks from the stakeholders, and sometimes tools or questionnaires. - Comprehensive assessment and notes review - We are currently running a clinic with a pharmacist and PA whereby de-prescribing is the focus - undertaking a QI project to measure outcome |
| 3.3 new developments | - availability of appropriate accommodation and support to manage people with challenging behaviour - Guidance to professionals on how it needs to be done: set targets as regards % of patients not yet rationalised alternatives to sedation: activities, better communication strategies, groups that enable peer supports for patients - The Cornwall BtC tool - need new tools - the Cornwall tool - I use the HONOS-LD - BtC connect - specialist STOMP pharmacists, access to behavioural/psychological help, supportive networks, including adequate social care support. - Offering social prescribing Access to day centres and day activities Access to nature, natural light and leisure activities like gym, holidays, .... - Train more clinicians in using and developing PBS. Make respite facilities more available to reduce carer burden. - ie those with schizophrenia who need maintenance antipsychotic prescribing - we can focus on dose reduction and augmentation with weight loss strategies etc. Relevent data to collect for this group will be antipsychotic(s), dose, weight, lipids, BP, hBA1c, if augmentation with aripiprazole/melatonin/GLP1 drugs are being used - which can be meaningfully evaluated looking for reductions in weight and look to reduce prescriptions of olanzapine and boost prescriptions of drugs with less chance of elevated hBA1c / weight For those with schizoaffective/bipolar we can collect data on number of agents used, which agents and their doses, the same biometrics as above and other markers such as sodium levels (multiple anti-epileptics cause hyponatraemia), EGFR if on lithium etc. The focus of this population can then be to try and reduce the number of agents being used, reduce use of drugs that can impair cognition (ie valproate and carbamazepine) and promote the use of newer drugs with less side effects (Aripiprazole, Lamotrigine, Lurasidone). For those with depression / anxiety disorders the focus could be on reducing the use of weight promoting drugs such as mirtazapine etc. And lastly, it may be worth considering the anticholinergic burden of drugs in those with learning disability certainly who are older. Reducing this can have a big impact on cognition for some. - Improve PBS presence and improve access to MHLD - A more robust understanding and implementation of PBS at a primary care level, more investment in care provision. - Use of PBS plans, - MDT approach. Confidence of carers and MDT in utilising PBS approaches - Looking at alternatives - Have PBS or alternative plans in place with skilled care providers before any implementation - Need to have alternatives/systems/resources/training in place to support the challening behaviour that psychotropic medication was prescribed for. - by having Psychology/Behaviour support input early on i.e. in CAMHS as at present a lot of people are started on antipsychotics in CAMHS without having had any input from psychology/BSS! - Greater access to psychology More community nursing to provide support - Identify any causal or aggravating factors which may benefit from non-pharm intervention. - Easier access to physical health care. |

| Themes | Participant comments (non-psychiatry) |
| --- | --- |
| 1. Breaking down barriers: Addressing systemic, structural, and resource challenges | |
| 1.1 staff issues | - ght community placements and staff turn over etc - services reducing and social care workforce that is not skilled enough - Difficulty with support staff who largely support the patients, they often are against reducing medication and request an increase in them once they have reduced as the patients can sometimes show difficult/challenging behaviours which can be managed but support staff find this hard. They likely need more support around this. - competence of those paid providers who support those special populations |
| 1.2 instability in community settings | - hard to withdraw meds when people are struggling to be in stable settings |
| 1.3 Non- pharmacological interventions | - There is a real challenge with providing non-pharmacological interventions that are sustainable with infrastructure of learning disability services reducing and social care workforce that is not skilled enough and environments that are not capable. - and environments that are not capable. |
| 1.4 financial constraints in staffing | - Yes we do see a significant changes in behaviour but not for all. Reduction can impact on peoples mental and challenging needs even though a good PBS is in place. Environment and not having financial facility to increase staffing can impact immensely - Perhaps time to embed - limited resource - Environment and not having financial facility to increase staffing can impact immensely |
| 1.5 Resistance to change | - parental and care staff resistance - I face opposition when mentioning a reduction of psychotropics with some ethnic minority families. This is usually as a result of their understanding of autism/learning disabilites. - Sadly I have experienced situations where parents have been extremely reluctant to have medication reduced and are often asking for more medication to be prescribed. |
| 1.6 Timing | - feel the overall drive is good, however, feel there can be over attempts for some teams/people to implement STAMP/STOP where it is not actually the right time. |
| 1.7 Impact of medication reduction on family | - People with a severe learning disability and in particular those prescribed first generation antipsychotics. The reduction of antipsychotic in this cohort often leads to alleviation of sedation that can be perceived as challenging for carers and/or family. - People with a severe learning disability and in particular those prescribed first generation antipsychotics. The reduction of antipsychotic in this cohort often leads to alleviation of sedation that can be perceived as challenging for carers and/or family. |
| 1.8 cultural barriers | - We still find minority groups less willing to accept the kind of holistic care required to move away from medication, often because of an unwillingness to join groups that may include other families from their cultural group. - Families of ethnic monitories appear to see medication as the answer to fix behaviour and appear unaware that through support and using different strategies that they can make positive changes of their child's behaviour. They appear to want a quick fix and seem to need more input around understanding the needs of their child and their behaviour. - Some people from ethnic minority backgrounds have:- - Language barriers - Difficulties with translation and/or willingness to engage with services. - when working with individuals who have immigrated to the UK, not enough is done to support the cultural change and behaviours can be seen as psychological rather than exposure to a new culture/language/home. |
| 1.9 MDT input | - Education sessions to MDT and patients and caters - Is there a review of need mapped onto a MDT supported biopsychosocial formulation. 3. Has education been given to person and their supporters re benefits, risks and opportunities of medication optimization? - Greater collaboration from local authority to address social factors - good use of MDT approach. - I am not qualified to answer this question - in my opinion we have a massive disconnect between professionals in the NHS which prevents an holistic approach to cutomer treatment and care. A customers physical health impacts there mental health massivly so why are consultants such as cardiologists etc not involved in MDT's regarding perseved mental health issues when it could be a side effect of medication or the physical condition. I believe to be fully effective as an MDT everyone involved in the customers care should attend any MDT meeting to ensure we have the whole picture. As an example I supported a customer who suddenly began over spending and being very aggressive to previously preferred staff - it turned out this was a side effect of her medication for a medical condition, this was only discovered through staff research since the consultant was not invited to MDT meetings regarding the behaviour. I strongly believe everyone involved in a customers care from the social worker (who has often never met the customer) to the GP to every consulatant/specialist or there rep needs to attend MDT meetings regardless of the issue to rule out other causes of behaviour/ concerns about mental health. i strongly believe this would reduce the over prescribing to the learning disability population. - Adequate and competent support packages to implement MDT recommendations - Really good history taking Appropriate MDT consultation and advice rather than psychiatry acting in isolation - Increased follow up intensive support from an MDT for an agreed period of time more psychological resources - more MDT work - Sustainability and future proofing STOMP teams. Co- creation MDT reduction Plans. - engagement of the wider MDT in managing STOMP patients. - A dedicated team approach with weekly STOMP/STAMP clinics where it will be easier to collect data and measure effectiveness. - Ensure all health checks have been completed. To consider least restrictive options and to weigh that up against risk. Discussion's where appropriate with MDT members to support, assess and monitor. To ensure adequate support is in place for patient. - Better MDT decision making. CORE LD scores or other outcome measures - Having a robust MDT approach to supporting those that are at risk/currently prescribed psychotropic medication - Execellent proactive MDT commitment - Consistent clinicians and MDT including primary care so that everyone understands what is happening and can support. Pt and carers are at the heart of this and should set the pace. - Education sessions to MDT and patients and caters - capable workforce without lengthy wait times for care - Specialist pharmacist input and dedicated workforce - MDT working - use MDT working and do not prescribe in a vacuum - Is there a review of need mapped onto a MDT supported biopsychosocial formulation. - Pharmacist working as part of MDT or conducting specialist STOMP clinics. |
| 1.10 Clear pathways for medication reduction | - /clear pathways e.g. behaviour pathway/MH pathway with access to alternative evidence based intervention/ |
| 1.11 national funding and specialist support | - Make it part of a national funded workstream. Empower PCN or GP practice pharmacists to do it, with the support of specialists eg outreach LD specialist pharmacist. - Time and the right support to do the work eg PCN pharmacist, behaviour support - Appropriate accommodation, provision and access to relevant services, finance and regular assessment/reviews |
| 1.12 quality of life measures | - Question 11 did not mention improved quality of life and overall happiness when people are better able to interact with their world which would have been my number 1 benefit. I - use existing QoL measures to assist in supporting people with their MH |
| 1. Implementing STOMP in ethnic minorities | |
| 2.1 Over reliance on medication | - For some people there is a preference for the medical model as a way of treating behaviour and mental health issues rather than a biopsychosocial formulation driven approach - Cultural differences and beliefs in some ethnic minorities support more of a medical model and the notion that medication will "fix" a person. This narrative can be difficult to shift. - Medication is often seen as the quick fix to problems. - Families of ethnic monitories appear to see medication as the answer to fix behaviour and appear unaware that through support and using different strategies that they can make positive changes of their child's behaviour. They appear to want a quick fix and seem to need more input around understanding the needs of their child and their behaviour. |
| 2.2 overprescribing | - We know in our service that ethnic minority clients are over-represented in our STOMP group - people who are taking psychotropic medications but who do not have a mental health diagnosis. I think the challenges lie in the lack of cultural understanding and flexibility of services - I don't find that there is an issue regarding ethnic populations - but GP's seem very happy to prescribe the learning disabilty populating anything and everything - on occassion myself or my team have had to raise issues around contraindiactions of the medication prescribed. |
| 2.3 cultural understanding | - I think the challenges lie in the lack of cultural understanding and flexibility of services. |
| 2.4 medication management and prescribing | - I have noticed that there is more over prescribing with ethnic minority groups. I have reflected on this and I think there are lots of reasons: sometimes there is an overvalue from the network on medication as opposed to other approaches/sometimes I think there is a tendency to see MH need when there is not and investment in thinking about the communicative value of behaviour is lost - I have noticed that there is more over prescribing with ethnic minority groups. I have reflected on this and I think there are lots of reasons: - sometimes there is an overvalue from the network on medication as opposed to other approaches/sometimes I think there is a tendency to see MH need when there is not and investment in thinking about the communicative value of behaviour is lost - It is difficult to engage with some of our ethnic minority patients/families. Within my role we have ensured to keep a data base of ethnic minority patients prescribed Psychotropic medication. |
| 2.5 medication reduction | - Risk aversion Tendency for deterioration immediately to be blamed on lack of / reduction in medication. Tendency for system to pivot to a medical response to crisis. - Health inequalities and unwillingness to change (of general adult psychiatrists who see people with learning disabilities and/or autism in non-specialist secondary MH services) - there is strong support in some clients and carers for the use of medication to treat distress and associated behavioural symptoms - sometimes clients have been on medication for many years and settled so there is fear of change and the impact of meds reduction – - Risk aversion - Reluctance of GPs and some consultant psychiatrists to change or remove psychotropic medications introduced by previous medics. - taff concerns around challenging behaviour when discussing reduction of medication. - Reduced engagement with non pharmacological support for behaviours of concern - poor quality of community provision in implementing other strategies to manage behaviour etc that means medication appears required - Circumstances which result in challenges in reducing antipsychotics for behaviours of concern - Tendency for deterioration immediately to be blamed on lack of / reduction in medication. |
| 1. Training and development of non-pharmacological approaches | |
| 3.1 outcome measures | - Outcome measures - Is there baseline data re behaviours/distress. - Outcome measures used to consider the impact of these approaches and psychological intervention on quality of life and engagement. - Good Quality Medication reviews. |
| 3.2 new developments | - ingle case design of introducing PBS/Trauma informed care or mental health psycho social supports – - PBS - Promote and offer alternative therapies such as PBS. - Staff /carer training re medication and PBS / formulation to improve confidence and understanding - Focus on behavioural intervention such as PBS assessment/plans; - Evidence of good positive behaviour support, prescribers taking into account the views and observations of the support staff who know the person well, timely medication reviews with the appropriate prescriber who has knowledge of STOMP. You can measure the impact of these measures by seeing a reduction in the medications prescribed or being given as a PRN and an increase in people's activities and quality of life through feedback from the person or those closest to them. - and clear strategies for alternatives to medication including PBS approaches e.g. life and coping skill development - Positive Behaviour Support - Positive Behavioural Support (PBS). Reduction in use of PRN use to manage challenging behaviours would allow measurement - PBS Good commissioning - Thorough, MDT implementation of PBS - THE use of PBS enviromental changes housing available to individuals who challenge - More training in PBS, trauma-informed care and other therapeutic approaches in the wider MDT, primary care, in education settings, social care settings, for providers etc. - Applying principles of PBS and/or other therapeutic approaches to support meeting the individuals needs as an alternative to medications. - Positive behavioural support. Families and services having better understanding of behaviour and using all of the non-pharmaceutical strategies - PBS embedded in culture of LD social care support in a genuine way (not tokenistic). This would improve the likelihood of alternative approaches being effectively used. Could be measured by audit of PBS plans underpinned by full functional assessment. - Use of therapies intervention, PBS plan and environmental modifications as first line treatment, - Within a framework of a comprehensive Positive Behaviour Support (PBS) approach - Quick easy access to positive behaviour support and appropriate carer support - The adoption of a therapeutic and holistic approach. Focus on trauma-informed care and communication. - Increase in use of quality PBS plan - improved understanding and implementation of Positive Behaviour Support by social care providers - PBS work and understanding less importance given the psychiatrist/medical prescriber as being the 'expert' or most knowledgeable in any system skilling up (knowledge, training and confidence) of health staff, social care and are staff at all levels - appropriate assessments have been carried out beforehand i.e capable environments, functional analysis, Positive behavioural support plan, communication assessments and sensory assessments. - Education sessions to MDT and patients and caters - improved education and support for family/carers. - Support and education to families about how we could reduce meds - Support and education to for Patients Families, Carers and Care providers to reduce anxieties around reduction. - Education for patients, carers and families |

| Themes | Participant comments (psychiatry) |
| --- | --- |
| the top THREE key barriers to reducing over or inappropriate psychotropic use in learning disability, autism, or both | |
| Unclear clinical responsibility for ongoing prescribing | - Unclear clinical responsibility for ongoing prescribing - Unclear clinical responsibility for ongoing prescribing - Unclear clinical responsibility for ongoing prescribing; - Unclear clinical responsibility for ongoing prescribing - Unclear clinical responsibility for ongoing prescribing - Unclear clinical responsibility for ongoing prescribing - Unclear clinical responsibility for ongoing prescribing - Unclear clinical responsibility for ongoing prescribing - Unclear clinical responsibility for ongoing prescribing - Unclear clinical responsibility for ongoing prescribing |
| Psychiatrist capacity | - Psychiatrist capacity - Psychiatrist capacity - Psychiatrist capacity; - Psychiatrist capacity; - Psychiatrist capacity; - Psychiatrist capacity - Psychiatrist capacity - Psychiatrist capacity; - Psychiatrist capacity - Psychiatrist capacity - Psychiatrist capacity - Psychiatrist capacity - Psychiatrist capacity - Psychiatrist capacity - Psychiatrist capacity;Psychiatrist confidence - Psychiatrist capacity - Psychiatrist capacity;Psychiatrist confidence - Psychiatrist confidence - Psychiatrist capacity |
| Primary care clinician capacity | - Primary care clinician capacity; - Primary care clinician confidence; - Primary care clinician confidence; - Confidence & capacity with primary care physicians. - Primary care clinician confidence; - Primary care clinician capacity; - Primary care clinician capacity; - Primary care clinician confidence - Primary care clinician confidence; - Primary care clinician confidence; - Primary care clinician confidence; - Primary care clinician confidence; |
| Lack of prioritization | - Not a priority - Not a priority/focus; - Not a priority/focus; - Not a priority/focus; |
| Lack of effective MDT approach | - Lack of effective MDT approach - Lack of effective MDT approach - Lack of effective MDT approach - Lack of effective MDT approach - Lack of effective MDT approach - Lack of effective MDT approach - Lack of effective MDT approach - Lack of effective MDT approach - Lack of effective MDT approach - Lack of effective MDT approach - Lack of effective MDT approach - Lack of effective MDT approach - Lack of effective MDT approach - Lack of effective MDT approach - Lack of effective MDT approach - Lack of effective MDT approach - Lack of effective MDT approach - Lack of effective MDT approach - Lack of effective MDT approach - Lack of effective MDT approach - Lack of effective MDT approach - Lack of effective MDT approach - Lack of effective MDT approach - Lack of effective MDT approach - Lack of effective MDT approach - Lack of effective MDT approach - Lack of effective MDT approach - Lack of effective MDT approach - Lack of effective MDT approach - Lack of effective MDT approach - Lack of effective MDT approach - Lack of effective MDT approach - Lack of effective MDT approach - Lack of mdt access and social opportunities - Lack of mdt access and social opportunities - Lack of mdt access and social opportunities |
| Lack of deprescribing guidance | - Lack of deprescribing guidance - Lack of deprescribing guidance - Lack of deprescribing guidance - Lack of deprescribing guidance - Lack of deprescribing guidance - Lack of deprescribing guidance - Lack of deprescribing guidance - Lack of deprescribing guidance - Lack of deprescribing guidance - Lack of deprescribing guidance - Lack of deprescribing guidance - Lack of deprescribing guidance - Lack of deprescribing guidance |
| Lack of access to alternatives to medication | - Lack of access to alternatives to medication; - Lack of access to alternatives to medication; - Lack of access to alternatives to medication; - Lack of access to alternatives to medication; - Lack of access to alternatives to medication; - Lack of access to alternatives to medication; - Lack of access to alternatives to medication; - Lack of access to alternatives to medication; - Lack of access to alternatives to medication; - Lack of access to alternatives to medication; - Lack of access to alternatives to medication; - Lack of access to alternatives to medication; - Lack of access to alternatives to medication; - Lack of access to alternatives to medication; - Lack of access to alternatives to medication; - Lack of access to alternatives to medication; - Lack of access to alternatives to medication; - Lack of access to alternatives to medication; - Lack of access to alternatives to medication; - Lack of access to alternatives to medication; - Lack of access to alternatives to medication; - Lack of access to alternatives to medication; - Lack of access to alternatives to medication; - Lack of access to alternatives to medication; - Lack of access to alternatives to medication; - Lack of access to alternatives to medication; - Lack of access to alternatives to medication; - Lack of access to alternatives to medication; - Lack of access to alternatives to medication; - Lack of access to alternatives to medication; - Lack of access to alternatives to medication; - Lack of access to alternatives to medication; - Lack of access to alternatives to medication; - Lack of access to alternatives to medication; - Lack of access to alternatives to medication; - Lack of access to alternatives to medication; - Lack of access to alternatives to medication; - Lack of access to alternatives to medication; - Lack of access to alternatives to medication; - Lack of access to alternatives to medication; - Lack of access to alternatives to medication; - Lack of access to alternatives to medication; |
| Family or carers preference | - Family or carers preference; - Family or carers preference; - Family or carers preference; - Family or carers preference; - Family or carers preference; - Family or carers preference; - Family or carers preference; - Family or carers preference; - Family or carers preference; - Family or carers preference; - Family or carers preference; - Family or carers preference; - Family or carers preference; - Family or carers preference; - Family or carers preference; - Family or carers preference; - Family or carers preference; - Family or carers preference; - Family or carers preference; - Family or carers preference; - Family or carers preference; - Family or carers preference; - Family or carers preference; - Family or carers preference; - Family or carers preference; - Family or carers preference; - Family or carers preference; - Family or carers preference; - Family or carers preference; - Family or carers preference; - Family or carers preference; - Family or carers preference; - Family or carers preference; - Family or carers preference; - Family or carers preference; - Family or carers preference; - Family or carers preference; - Family or carers preference; - Family or carers preference; - Family or carers preference; - Family or carers preference; - Family or carers preference; - Family or carers preference; - Family or carers preference; - Family or carers preference; - Family or carers preference; - Family or carers preference; - Family or carers preference; - Family or carers preference; - Family or carers preference; - Family or carers preference; |
| Other Barrier(s) | |
| STOMP negative consequence | - Another important barrier is the fact that there is little recognition in the national programme that a lot of harm can be done with STOMP approaches (which I think reflects most clinicians experiences hence many don't engage well with the programme). If you unmask schizophrenia when an antipsychotic is stopped then you can have a stepwise decline in function that doesn't recover and often the patient will end up on a higher dose of antipsychotic to treat the relapse. - Risk of with drawl - and need for inpatient care. - Lack of suitable inpatient beds to admit in crisis i.e. if medication reduction results in increased physical aggression, self-injurious behaviours etc. Unless there is access locally to specialist LD beds the implementation of STOMP is unrealistic as it could result in a Serious Incident. - Fear of destabilisation - Concern from carers (non family) as to what would happen if medication is reduced. |
| Lack of support | - Since the switch to electronic notes it can be difficult to get a good long term history - and this can lead to reduced medications (which were thought to be for behavioural control) leading to relapse of a long term mental health condition about which early history had been lost. - Lack of timely social care input, - Social care providing sufficient funding for care. Main reason for prescribing is to manage lack of social care input I.e. chemical restraint. - Effective care providers and social care input including provision of adequate day services, respite and activities. - System around the patient, i.e. effective and supportive social care - with consistent carers. Huge impact upon our patients. - lack of staff support funding/training - Lack of appropriate respite and activity provision outside of school is also a factor in this. - Access to respite, access to day services, access to meaningful daytime activity - There is a significant issue regarding the understanding as to the original reasons for prescription; you also don't have pharmacy input within the options above as, within our CTLD, we have a pharmacy lead with psychiatry support and it is a very effective model. - lack of pharmacist support - There is a significant issue regarding the understanding as to the original reasons for prescription; you also don't have pharmacy input within the options above as, within our CTLD, we have a pharmacy lead with psychiatry support and it is a very effective model. - Yes - lack of capacity within MDT to create PBS plans - Lack of Psychologists inputs eg no sensory integration assessment, Lack of BSP in place, low carer : patient ratio. - Lack of timely social care input, capability of community provider and environment - Inappropriate environme - e family unit being unable to provide the level of adjustments the child needs in the context of the needs of their other children and so medicaiton being needed to manage risks associated with extreme meltdowns etc which could perhaps be avoided in a setting where the child could have undivided adult attention. - Social care providing sufficient funding for care. Main reason for prescribing is to manage lack of social care input I.e. chemical restraint - capability of community provider and environment |
| Diagnostic overshadowing | - Underlying mental illness aka discovering that it was not overprescribing but the person had been known for a long time and the initial presentation was not recorded appropriately - The biggest barrier in my view is lack of diagnostic clarity for what medications may be prescribed for. There are many patients with no diagnoses other that Learning Disability on a cocktail of medications and when you review their notes in detail you see that they are likely being treated for the common comorbid disorders such as OCD, schizophrenia, Bipolar and in older patients often ASD/ADHD may be a factor. Another important barrier is the fact that there is little recognition in the national programme that a lot of harm can be done with STOMP approaches (which I think reflects most clinicians experiences hence many don't engage well with the programme). If you unmask schizophrenia when an antipsychotic is stopped then you can have a stepwise decline in function that doesn't recover and often the patient will end up on a higher dose of antipsychotic to treat the relapse. - The biggest barrier in my view is lack of diagnostic clarity for what medications may be prescribed for. There are many patients with no diagnoses other that Learning Disability on a cocktail of medications and when you review their notes in detail you see that they are likely being treated for the common comorbid disorders such as OCD, schizophrenia, Bipolar and in older patients often ASD/ADHD may be a factor. |

| Themes | Participant comments (non-psychiatry) |
| --- | --- |
| the top THREE key barriers to reducing over or inappropriate psychotropic use in learning disability, autism, or both | |
| Unclear clinical responsibility for ongoing prescribing | - Unclear clinical responsibility for ongoing prescribing - Unclear clinical responsibility for ongoing prescribing - Unclear clinical responsibility for ongoing prescribing - Unclear clinical responsibility for ongoing prescribing - Unclear clinical responsibility for ongoing prescribing - Unclear clinical responsibility for ongoing prescribing - Unclear clinical responsibility for ongoing prescribing - Unclear clinical responsibility for ongoing prescribing - Unclear clinical responsibility for ongoing prescribing - Unclear clinical responsibility for ongoing prescribing - Unclear clinical responsibility for ongoing prescribing - Unclear clinical responsibility for ongoing prescribing - Unclear clinical responsibility for ongoing prescribing - Unclear clinical responsibility for ongoing prescribing - Unclear clinical responsibility for ongoing prescribing - Unclear clinical responsibility for ongoing prescribing - Unclear clinical responsibility for ongoing prescribing - Unclear clinical responsibility for ongoing prescribing - Unclear clinical responsibility for ongoing prescribing - Unclear clinical responsibility for ongoing prescribing - Unclear clinical responsibility for ongoing prescribing - Unclear clinical responsibility for ongoing prescribing - Unclear clinical responsibility for ongoing prescribing - Unclear clinical responsibility for ongoing prescribing - Unclear clinical responsibility for ongoing prescribing - Unclear clinical responsibility for ongoing prescribing - Unclear clinical responsibility for ongoing prescribing - Unclear clinical responsibility for ongoing prescribing - Unclear clinical responsibility for ongoing prescribing - Unclear clinical responsibility for ongoing prescribing - Unclear clinical responsibility for ongoing prescribing - Unclear clinical responsibility for ongoing prescribing - Unclear clinical responsibility for ongoing prescribing - Unclear clinical responsibility for ongoing prescribing - Unclear clinical responsibility for ongoing prescribing - Unclear clinical responsibility for ongoing prescribing - Unclear clinical responsibility for ongoing prescribing |
| Psychiatrist capacity | - Psychiatrist confidence - Psychiatrist confidence - Psychiatrist confidence - Psychiatrist confidence - Psychiatrist confidence - Psychiatrist confidence - Psychiatrist confidence - Psychiatrist confidence - Psychiatrist confidence - Psychiatrist confidence - Psychiatrist confidence - Psychiatrist confidence - Psychiatrist confidence - Psychiatrist confidence - Psychiatrist confidence - Psychiatrist capacity; - Psychiatrist capacity; - Psychiatrist capacity; - Psychiatrist capacity; - Psychiatrist capacity; - Psychiatrist capacity; - Psychiatrist capacity; - Psychiatrist capacity; - Psychiatrist capacity; - Psychiatrist capacity; - Psychiatrist confidence - Psychiatrist confidence - Psychiatrist confidence - Psychiatrist confidence - Psychiatrist confidence - Psychiatrist confidence - Psychiatrist confidence - Psychiatrist confidence - Psychiatrist confidence - Psychiatrist confidence - Psychiatrist confidence - lack of knowledge surrounding inappropriate medication use Difficulty in challenging psychiatrist prescribing - Psychiatrist confidence - Psychiatrist confidence - Psychiatrist confidence - Psychiatrist confidence - Psychiatrist confidence - Psychiatrist confidence - Psychiatrist confidence - Psychiatrist confidence - Psychiatrist confidence - Psychiatrist confidence - Psychiatrist confidence - Psychiatrist confidence - Psychiatrist confidence - Psychiatrist capacity; - Psychiatrist capacity; - Psychiatrist capacity; - Psychiatrist capacity; - Psychiatrist capacity; |
| Primary care clinician capacity | - Primary care clinician confidence; - Primary care clinician confidence; - Primary care clinician confidence; - Primary care clinician confidence; - Primary care clinician confidence; - Primary care clinician confidence; - Primary care clinician confidence; - Primary care clinician confidence; - Primary care clinician confidence; - Primary care clinician confidence; - Primary care clinician confidence; - Primary care clinician confidence; - Primary care clinician confidence; - Primary care clinician confidence; - Primary care clinician confidence; - Primary care clinician confidence; - Primary care clinician confidence; - Primary care clinician confidence; - Primary care clinician confidence; - Primary care clinician confidence; - Primary care clinician confidence; - Primary care clinician confidence; - Primary care clinician confidence; - Primary care clinician confidence; - Primary care clinician confidence; - Primary care clinician confidence; - Primary care clinician confidence; - Primary care clinician confidence; - Primary care clinician confidence; - Primary care clinician confidence; - Primary care clinician confidence; - Primary care clinician confidence; - Primary care clinician confidence; - Primary care clinician confidence; - Primary care clinician confidence; - Primary care clinician confidence; - Primary care clinician confidence; - Primary care clinician confidence; - Primary care clinician confidence; - Primary care clinician confidence; - Primary care clinician confidence; - Primary care clinician confidence; - Primary care clinician confidence; - Primary care clinician confidence; - Primary care clinician confidence; - Primary care clinician confidence; - Primary care clinician confidence; - Primary care clinician confidence; - Primary care clinician confidence; - Primary care clinician confidence; - Primary care clinician confidence; - Primary care clinician confidence; - Primary care clinician confidence; - Primary care clinician confidence; - Primary care clinician confidence; - Primary care clinician confidence;Primary care clinician capacity - Primary care clinician confidence;Primary care clinician capacity - Primary care clinician confidence;Primary care clinician capacity - Primary care clinician confidence;Primary care clinician capacity |
| Lack of prioritization | - Not a priority/focus; - Not a priority/focus; - Not a priority/focus; - Not a priority/focus; - Not a priority/focus; - Not a priority/focus; - Not a priority/focus; - Not a priority/focus; - Not a priority/focus; - Not a priority/focus; - Not a priority/focus; - Not a priority/focus; - Not a priority/focus; - Not a priority/focus; - Not a priority/focus; - Not a priority/focus; - Not a priority/focus; - Not a priority/focus; - Not a priority/focus; - Not a priority/focus; - Not a priority/focus; - Not a priority/focus; - Not a priority/focus; - Not a priority/focus; - Not a priority/focus; - Not a priority/focus; - Not a priority/focus; - Not a priority/focus; - Not a priority/focus; - Not a priority/focus; |
| Lack of effective MDT approach | - Lack of effective MDT approach; - Lack of effective MDT approach; - Lack of effective MDT approach; - Lack of effective MDT approach; - Lack of effective MDT approach; - Lack of effective MDT approach; - Lack of effective MDT approach; - Lack of effective MDT approach; - Lack of effective MDT approach; - Lack of effective MDT approach; - Lack of effective MDT approach; - Lack of effective MDT approach; - Lack of effective MDT approach; - Lack of effective MDT approach; - Lack of effective MDT approach; - Lack of effective MDT approach; - Lack of effective MDT approach; - Lack of effective MDT approach; - Lack of effective MDT approach; - Lack of effective MDT approach; - Lack of effective MDT approach; - Lack of effective MDT approach; - Lack of effective MDT approach; - Lack of effective MDT approach; - Lack of effective MDT approach; - Lack of effective MDT approach; - Lack of effective MDT approach; - Lack of effective MDT approach; - Lack of effective MDT approach; - Lack of effective MDT approach; - Lack of effective MDT approach; - Lack of effective MDT approach;\ Lack of effective MDT approach; - Lack of effective MDT approach; - Lack of effective MDT approach; - Lack of effective MDT approach; - Lack of effective MDT approach; - Lack of effective MDT approach; - Lack of effective MDT approach; - Lack of effective MDT approach; - Lack of effective MDT approach; - Lack of effective MDT approach; - Lack of effective MDT approach; - Lack of effective MDT approach; - V - Lack of effective MDT approach; - Lack of effective MDT approach; - Lack of effective MDT approach; - Lack of effective MDT approach; - Lack of effective MDT approach; - Lack of effective MDT approach; - Lack of effective MDT approach; - Lack of effective MDT approach; - Lack of effective MDT approach; - Lack of effective MDT approach; - Lack of effective MDT approach; - Lack of effective MDT approach; - Lack of effective MDT approach; - Lack of effective MDT approach; - Lack of effective MDT approach; - Lack of effective MDT approach; - Lack of effective MDT approach; - Lack of effective MDT approach; - Lack of effective MDT approach; - Lack of effective MDT approach; - Lack of effective MDT approach; - Lack of effective MDT approach; - Lack of effective MDT approach; - Lack of effective MDT approach; - Lack of effective MDT approach; - Lack of effective MDT approach; - Lack of effective MDT approach; - Lack of effective MDT approach; - Lack of effective MDT approach; |
| Lack of deprescribing guidance | - Lack of deprescribing guidance; - Lack of deprescribing guidance; - Lack of deprescribing guidance; - Lack of deprescribing guidance; - Lack of deprescribing guidance; - Lack of deprescribing guidance; - Lack of deprescribing guidance; - Lack of deprescribing guidance; - Lack of deprescribing guidance; - Lack of deprescribing guidance; - Lack of deprescribing guidance; - Lack of deprescribing guidance; - Lack of deprescribing guidance; - Lack of deprescribing guidance; - Lack of deprescribing guidance; - Lack of deprescribing guidance; - Lack of deprescribing guidance; - Lack of deprescribing guidance; - Lack of deprescribing guidance; - Lack of deprescribing guidance; - Lack of deprescribing guidance; - Lack of deprescribing guidance; - Lack of deprescribing guidance; - Lack of deprescribing guidance; - Lack of deprescribing guidance; - Lack of deprescribing guidance; - Lack of deprescribing guidance; - Lack of deprescribing guidance; - Lack of deprescribing guidance; |
| Lack of access to alternatives to medication | - Lack of access to alternatives to medication; - Lack of access to alternatives to medication; - Lack of access to alternatives to medication; - Lack of access to alternatives to medication; - Lack of access to alternatives to medication; - Lack of access to alternatives to medication; - Lack of access to alternatives to medication; - Lack of access to alternatives to medication; - Lack of access to alternatives to medication; - Lack of access to alternatives to medication; - Lack of access to alternatives to medication; - Lack of access to alternatives to medication; - Lack of access to alternatives to medication; - Lack of access to alternatives to medication; - Lack of access to alternatives to medication; - Lack of access to alternatives to medication; - Lack of access to alternatives to medication; - Lack of access to alternatives to medication; - Lack of access to alternatives to medication; - Lack of access to alternatives to medication; - Lack of access to alternatives to medication; - Lack of access to alternatives to medication; - Lack of access to alternatives to medication; - Lack of access to alternatives to medication; - Lack of access to alternatives to medication; - Lack of access to alternatives to medication; - Lack of access to alternatives to medication; - Lack of access to alternatives to medication; - Lack of access to alternatives to medication; - Lack of access to alternatives to medication; - Lack of access to alternatives to medication; - Lack of access to alternatives to medication; - Lack of access to alternatives to medication; - Lack of access to alternatives to medication; - Lack of access to alternatives to medication; - Lack of access to alternatives to medication; - Lack of access to alternatives to medication; - Lack of access to alternatives to medication; - Lack of access to alternatives to medication; - Lack of access to alternatives to medication; - Lack of access to alternatives to medication; - Lack of access to alternatives to medication; - Lack of access to alternatives to medication; - Lack of access to alternatives to medication; - Lack of access to alternatives to medication; - Lack of access to alternatives to medication; - Lack of access to alternatives to medication; - Lack of access to alternatives to medication; - Lack of access to alternatives to medication; - Lack of access to alternatives to medication; - Lack of access to alternatives to medication; - Lack of access to alternatives to medication; - Lack of access to alternatives to medication; - Lack of access to alternatives to medication; - Lack of access to alternatives to medication; - Lack of access to alternatives to medication; - Lack of access to alternatives to medication; - Lack of access to alternatives to medication; - Lack of access to alternatives to medication; - Lack of access to alternatives to medication; - Lack of access to alternatives to medication; - Lack of access to alternatives to medication; - Lack of access to alternatives to medication; - Lack of access to alternatives to medication; - Lack of access to alternatives to medication; - Lack of access to alternatives to medication; - Lack of access to alternatives to medication; - Lack of access to alternatives to medication; - Lack of access to alternatives to medication; - Lack of access to alternatives to medication; - Lack of access to alternatives to medication; - Lack of access to alternatives to medication; - Lack of access to alternatives to medication; |
| Family or carers preference | - Family or carers preference; - Family or carers preference; - Family or carers preference; - Family or carers preference; - Family or carers preference; - Family or carers preference; - Family or carers preference; - Family or carers preference; - Family or carers preference; - Family or carers preference; - Family or carers preference; - Family or carers preference; - Family or carers preference; - Family or carers preference; - Family or carers preference; - Family or carers preference; - Family or carers preference; - Family or carers preference; - Family or carers preference; - Family or carers preference; - Family or carers preference; - Family or carers preference; - Family or carers preference; - Family or carers preference; - Family or carers preference; - Family or carers preference; - Family or carers preference; - Family or carers preference; - Family or carers preference; - Family or carers preference; - Family or carers preference; - Family or carers preference; - Family or carers preference; - Family or carers preference; - Family or carers preference; - Family or carers preference; - Family or carers preference; - Family or carers preference; - Family or carers preference; - Family or carers preference; - Family or carers preference; - Family or carers preference; - Family or carers preference; - Family or carers preference; - Family or carers preference; - Family or carers preference; - Family or carers preference; - Family or carers preference; - Family or carers preference; - Family or carers preference; - Family or carers preference; - Family or carers preference; - Family or carers preference; - Family or carers preference; - Family or carers preference; - Family or carers preference; - Family or carers preference; - Family or carers preference; - Family or carers preference; - Family or carers preference; - Family or carers preference; - Family or carers preference; - Family or carers preference; - Family or carers preference; - Family or carers preference; - Family or carers preference; - Family or carers preference; - Family or carers preference; - Family or carers preference; - Family or carers preference; - Family or carers preference; - Family or carers preference; - Family or carers preference; - Family or carers preference; - Family or carers preference; - Family or carers preference; - Family or carers preference; - Family or carers preference; |
| Other Barrier(s) | |
| STOMP negative consequence | - staff concern/risk averse - Fear of change. Lot's of families have seen their loved ones suffer with either severe and frequent seizures or as a result of terrible and sometimes life changing side effects of medications in the past. If they feel that the current situation is stable they often do not want anything to be changed in case this happens again. - Concerns of impact on behaviours of concern due to length of time patient has been on medication. - Concerns of impact on behaviours of concern due to length of time patient has been on medication. - Competence of providers - Varied understandign and capacity in social care workforce - The absence of skilled providers, effective social care, robust and diverse community placements, fractured communities and families, non-existent early help (in adult services) all contribute to the fact that we cannot possibly provide the capable environments required to safely consider alternatives to medication. - Family or Paid carer ability to implement support plans that would manage the behaviour |
| Lack of support | - No funding for pharmacist's input. - Lack of effective medication monitoring and review - Reduction in paid social care support Lack of specialist care providers - ccess to a specialist pharmacist to deliver - Limited access to psychological and practical support - ack of skilled workforce who are able to support individuals effectively though a PBS approach - Lack of training/skill in PBS in the wider teams and provider level - Lack of appropriate social care package - lack of resources to implement alternative approaches (e.g. community support/ staffing) for more PBS type approaches. - Having the resources ie, - Lack of skilled and confident social care workforce - Social care/Housing |
| Diagnostic overshadowing | - A lack of knowledge around Learning Disabilities 2) A lack of Knowledge around Autism 3) Grouping Learning Disabilities and Autism in the same categories - situation I have encountered several occasions working in patients young person stable but prescribed multiple psychotropics- on account of need for social housing placement there is always a concern about any thing that would destabilise the individual so lack of drive to consider reducing unnecessary medication so concerning when some patients are discharge ready for 12-18 months before suitable placement found - I think we do not get the diagnostics right many of my patient s with a learning disability may also have autism or adhd and are not known or treated for appropriate conditions, instead get antipsychotics - Limited understanding of the functions that challenging behaviour may serve in people with learning disabilities and/or autism. - Pt's present with range of co-morbid and complex disorders |
