# Supplementary material for: Psychiatrists and non-psychiatrists’ attitudes to psychotropic optimisation for people with intellectual disabilities and/or autism: cross-sectional comparison study
Source: BJPsych Open. 2025 Oct 23;11(6):e249. doi: 10.1192/bjo.2025.10875 (PMC12569613; doi:10.1192/bjo.2025.10875)
Supplement: Tromans et al. supplementary material 3 — Tromans et al. supplementary material [file S2056472425108752sup003.docx]

Supplementary information 1: The survey question items.

| 1. Please select your job role. 2. Where in the country are you working? 3. How supportive are you of a national quality improvement programme that aims to reduce over and inappropriate prescribing of psychotropics in learning disability, autism, or both? 4. To what extent do you agree that it is possible to significantly (> 50%) reduce over or inappropriate prescribing of psychotropics in learning disability, autism, or both? 5. Do you encounter specific challenges when implementing STOMP/ STAMP in special populations particularly ethnic minorities? 6. If 'Yes' to the above question, give information below |
| --- |
| 1. When being prescribed for behaviour that challenges, order the psychotropic medicine groups in order of priority for focused work to reduce patient harm (1= top priority). Click on each option, drag a 2. How important is medicine optimisation of psychotropics for people with LD, autism or both? 3. What are the top THREE key barriers to reducing over or inappropriate psychotropic use in learning disability, autism, or both? (Other key barriers not mentioned in this list can be added in the next 4. Did you want other barrier(s) to have been offered in question 6 that you would have chosen in your top 3? (If 'No' go to next question. If 'Yes' provide details below) 5. What do you perceive as the key benefits to patients from reducing over or inappropriate prescribing of psychotropics. Please rank in order where 1 is the most important benefit? Click on each option, 6. In your experience what are the strategies that would help to rationalise psychotropic prescribing in people with LD, Autism or both.  How would you measure the impact of these? |
