# Supplementary material for: Psychiatrists and non-psychiatrists’ attitudes to psychotropic optimisation for people with intellectual disabilities and/or autism: cross-sectional comparison study
Source: BJPsych Open. 2025 Oct 23;11(6):e249. doi: 10.1192/bjo.2025.10875 (PMC12569613; doi:10.1192/bjo.2025.10875)
Supplement: Tromans et al. supplementary material 4 — Tromans et al. supplementary material [file S2056472425108752sup004.pdf]

Survey to understand views on a possible national medicines safety improvement programme

220 Responses 12:33 Average time to complete Closed Status

1. Do you agree to your data being used for this purpose?

Yes	219
No - Unfortunately, we cannot...	1

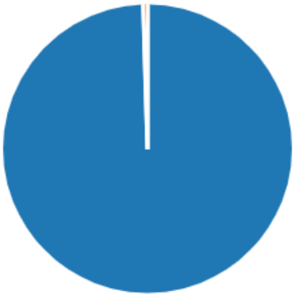

2. Please select your job role

Psychiatrist - not working with...	0
Psychiatrist - less than 5 years ...	12
Psychiatrist - 6 to 9 years exp...	15
Psychiatrist - 10 or more years...	39
Psychologist	23
Mental health nurse	4
Pharmacist	24
General practitioner	3
Mental health support worker	0
Learning disability nurse	59
Social care professional	10
Other	26

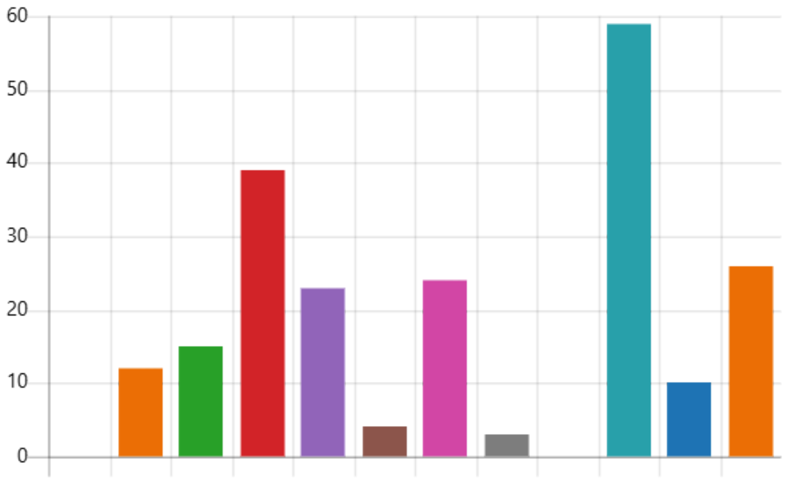

3. Where in the country are you working?

North West	23
North East	43
Midlands	49
London	29
South West	19
South East	18
Scotland	0
Wales	25
Other	16

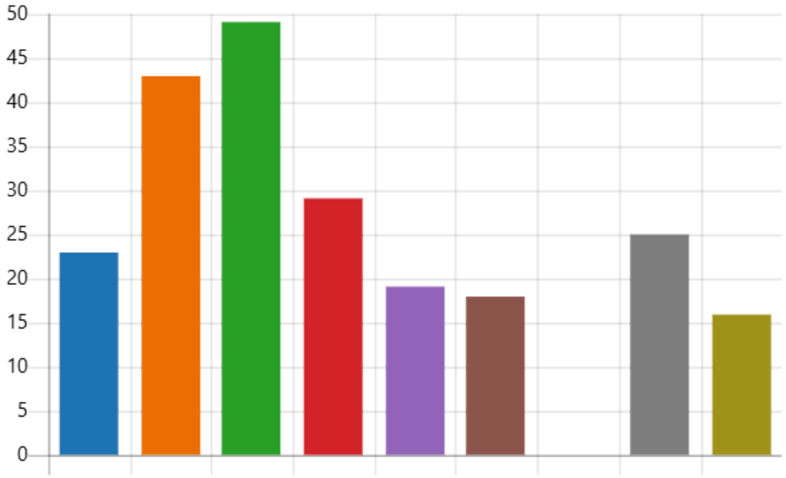

4. How supportive are you of a national quality improvement programme that aims to reduce over and inappropriate prescribing of psychotropics in learning disability, autism, or both?

Very supportive	154
Supportive	50
Unsure	13
Not supportive	0
Very unsupportive	1

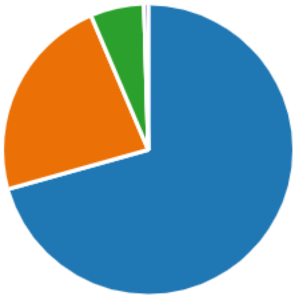

5. To what extent do you agree that it is possible to significantly (> 50%) reduce over or inappropriate prescribing of psychotropics in learning disability, autism, or both?

Promoters	45
Passives	85
Detractors	88

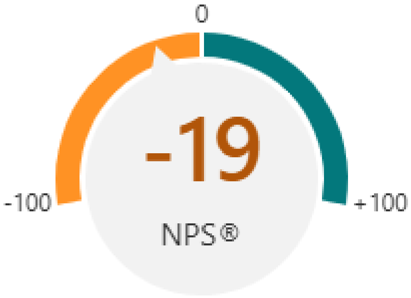

6. Do you encounter specific challenges when implementing STOMP/ STAMP in special populations particularly ethnic minorities?

Yes	66
No	62
Don't know	91

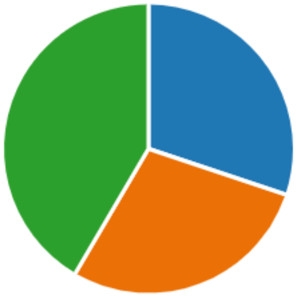

7. If 'Yes' to the above question, give information below

62
Responses

Latest Responses

"Sadly I have experienced situations where parents have b...

"Often started by specialist teams in local area with no su...

8. When being prescribed for behaviour that challenges, order the psychotropic medicine groups in order of priority for focused work to reduce patient harm (1= top priority). **Click on each option, drag and drop in a required order.**

Rank	Options
1	Antipsychotics
2	Combinations of psychotropics
3	Benzodiazepines
4	Anti-depressants
5	Hypnotics including Z drugs
6	Anti-seizure

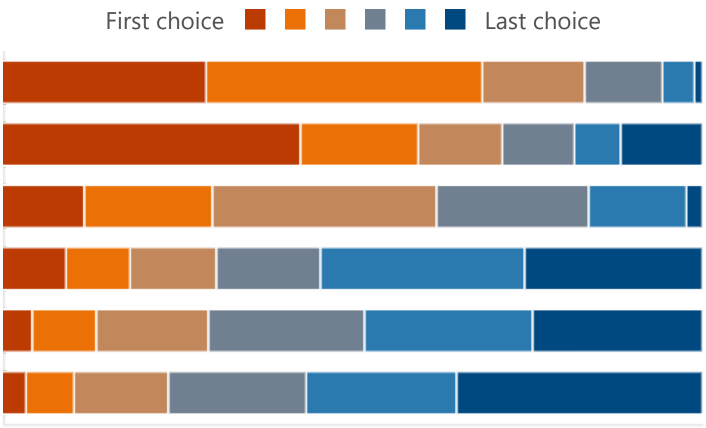

9. How important is medicine optimisation of psychotropics for people with LD, autism or both?

Extremely important	167
Somewhat important	38
Neutral	13
Somewhat not important	1
Extremely not important	0

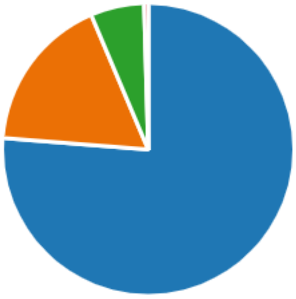

10. What are the top THREE key barriers to reducing over or inappropriate psychotropic use in learning disability, autism, or both? (Other key barriers not mentioned in this list can be added in the next question)

Psychiatrist capacity	46
Psychiatrist confidence	33
Primary care clinician capacity	19
Primary care clinician confiden...	54
Unclear clinical responsibility f...	49
Lack of access to alternatives t...	115
Lack of effective MDT approach	110
Lack of deprescribing guidance	42
Not a priority/focus	33
Patient preference	28
Family or carers preference	122

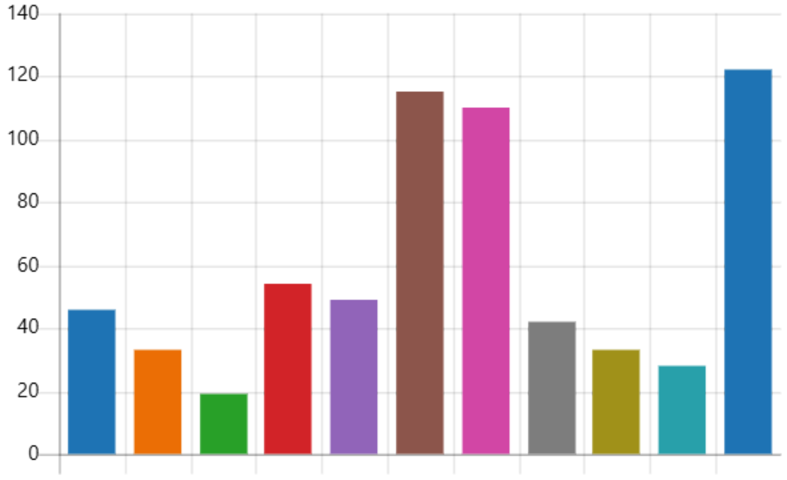

11. Did you want other barrier(s) to have been offered in question 6 that you would have chosen in your top 3? (If 'No' go to next question. If 'Yes' provide details below)

99
Responses

Latest Responses

"Concerns of impact on behaviours of concern due to leng...

"Yes unclear information given on starting therapy, no disc...

12. What do you perceive as the key benefits to patients from reducing over or inappropriate prescribing of psychotropics. Please rank in order where 1 is the most important benefit? **Click on each option, drag and drop in a required order.**

Rank	Options
1	Reduced risks to physical heal...
2	Reduced side-effects
3	Increased social activity
4	Increased physical activity

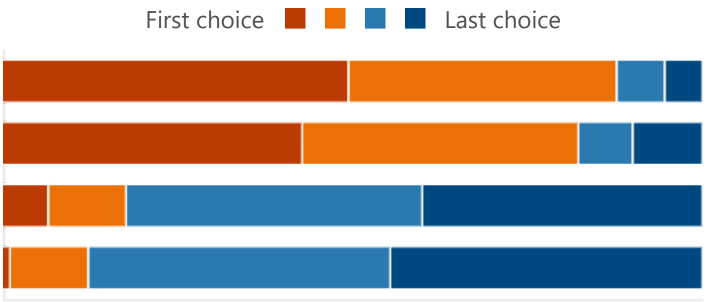

13. In your experience what are the strategies that would help to rationalise psychotropic prescribing in people with LD, Autism or both. How would you measure the impact of these?

164
Responses

Latest Responses

"Ensuring MDT approach, appropriate assessments have b...

"Focused projects "

"psycho education better access to other meds - like ADH...

Microsoft 365

Microsoft Forms | AI-Powered surveys, quizzes and polls [Create my own form](https://forms.office.com)

Privacy and cookies (https://go.microsoft.com/fwlink/?LinkId=521839) | Terms of use (https://go.microsoft.com/fwlink/?linkid=866263)
